# Long Term Delta-9-tetrahydrocannabinol Administration Inhibits Proinflammatory Responses in Minor Salivary Glands of Chronically Simian Immunodeficieny Virus Infected Rhesus Macaques

**DOI:** 10.3390/v12070713

**Published:** 2020-07-01

**Authors:** Xavier Alvarez, Karol Sestak, Siddappa N. Byrareddy, Mahesh Mohan

**Affiliations:** 1 Southwest National Primate Research Center, Texas Biomedical Research Institute, San Antonio, TX 78227, USA; jalvarezhernandez@txbiomed.org; 2 PreCliniTria, LLC., Mandeville, LA 70471, USA; ksestak.tulane@gmail.com; 3 Tulane National Primate Research Center, Covington, LA 70433, USA; 4 Department of Pharmacology and Experimental Neuroscience, University of Nebraska Medical Center, Omaha, NE 68198, USA

**Keywords:** THC, HIV/SIV, rhesus macaque, minor salivary gland inflammation, miR-29b

## Abstract

HIV/SIV-associated oral mucosal disease/dysfunction (HAOMD) (gingivitis/periodontitis/salivary adenitis) represents a major comorbidity affecting HIV patients on anti-retroviral therapy. Using a systems biology approach, we investigated molecular changes (mRNA/microRNA) underlying HAOMD and its modulation by phytocannabinoids (delta-9-tetrahydrocannabinol (∆^9^-THC)) in uninfected (*n* = 5) and SIV-infected rhesus macaques untreated (VEH-untreated/SIV; *n* = 7) or treated with vehicle (VEH/SIV; *n* = 3) or ∆^9^-THC (THC/SIV; *n* = 3). Relative to controls, fewer mRNAs were upregulated in THC/SIV compared to VEH-untreated/SIV macaques. Gene enrichment analysis showed differential enrichment of biological functions involved in anti-viral defense, Type-I interferon, Toll-like receptor, RIG-1 and IL1R signaling in VEH-untreated/SIV macaques. We focused on the anti-ER-stress anterior gradient-2 (*AGR2*), epithelial barrier protecting and anti-dysbiotic WAP Four-Disulfide Core Domain-2 (*WFDC2*) and glucocorticoid-induced anti-inflammatory *TSC22D3* (TSC22-domain family member-3) that were significantly downregulated in oropharyngeal mucosa (OPM) of VEH-untreated/SIV macaques. All three proteins localized to minor salivary gland acini and secretory ducts and showed enhanced and reduced expression in OPM of THC/SIV and VEH/SIV macaques, respectively. Additionally, inflammation associated miR-21, miR-142-3p and miR-29b showed significantly higher expression in OPM of VEH-untreated/SIV macaques. *TSC22D3* was validated as a target of miR-29b. These preliminary translational findings suggest that phytocannabinoids may safely and effectively reduce oral inflammatory responses in HIV/SIV and other (autoimmune) diseases.

## 1. Introduction

Although innate and adaptive immune functions are maintained in the oral mucosa during acute HIV/SIV infection, these responses become markedly impaired during chronic HIV infection and are not fully restored by combination anti-retroviral therapy [1,2,3,4,5,6]. Persistent oral mucosal inflammation/immune activation characterized by dysregulated proinflammatory cytokine production and dysbiosis are characteristics of chronic HIV/SIV infection [2,3,4,7,8,9,10,11,12]. Recent studies have identified moderate to severe oral manifestations (chronic periodontitis) in about 66% of HIV-infected patients on cART, suggesting persistent oral immune activation in cART treated patients [10]. 

Several studies have provided detailed descriptions of oral manifestations and their accompanying changes in immunophenotypic composition and cytokine production in HIV/SIV infection [1,2,3,4,8,9,11,12,13,14]. Nevertheless, the underlying molecular mechanisms remain unclear and represent an understudied area of HIV/SIV infection. In the past decade, miRNAs have emerged as powerful regulators of diverse cellular processes with more important roles in immune cell development, inflammatory responses and disease pathogenesis [15,16]. In addition to cytokines and transcription factors, recent studies show miRNA-mediated gene regulation to play critical roles in the pathogenesis of periodontitis (PO), Sjogren (Sjs), Oral lichen planus (OLP) and oral malignancy [17,18,19,20]. In this regard, a causal relationship between dysregulated miRNA (miR-146a, miR-30 and miR-155) expression and periodontitis has been proposed for further investigation [21,22,23]. However, it is striking that miRNA-mediated dysregulation of gene expression in the oral cavity during the course of HIV/SIV infection remains unknown and unaddressed.

While cART suppresses plasma viremia and partially restores immune reconstitution, chronic systemic and local immune activation (oral cavity) persists [2,3,4,7,9,10] and does not fully subside to baseline levels. Therefore, an important question with potential implication for oral immune homeostasis includes whether immune-modulating agents either alone or in combination with cART can reverse some of the oral immune perturbations induced by chronic HIV/SIV disease and whether feasible pharmacological strategies aimed at attenuating inflammation/immune cell activation can slow HIV/SIV disease progression, restore oral immune function and prevent further loss of immune function. 

Although we demonstrated the anti-inflammatory effects of cannabinoids in the distal gastrointestinal (GI) tract (colon and jejunum) of SIV-infected rhesus macaques [24,25,26], its impact on the upper GI tract, especially on HIV/SIV-induced oral mucosal inflammation, remained to be determined. Here, we report the first genome wide changes in gene and microRNA expression in the oropharyngeal mucosa in response to chronic SIV infection and more importantly, demonstrate the potential of long-term administration of the anti-inflammatory cannabinoid, delta-9-tetrahydrocannabinol (∆^9^-THC) to effectively modulate proinflammatory gene expression. Specifically, long-term THC administration preserved the expression levels of *AGR2* (anterior gradient 2)*, WFDC2* (WAP Four-Disulfide Core Domain 2) and *TSC22D3* (TSC22 domain family member 3), three important proteins that play key roles in maintenance of the structure and function of the oral mucosal barrier. Furthermore, we demonstrate the ability of miR-29b, a miRNA significantly upregulated in oropharyngeal mucosa (OPM) of SIV-infected rhesus macaques, to post-transcriptionally regulate *TSC22D3* expression. To the best of our knowledge, these preliminary findings for the first time highlight the anti-inflammatory effects of cannabinoids in the oral cavity during HIV/SIV infection. These findings have broader implications for not only HIV but also other chronic inflammatory diseases of the minor salivary gland (MiSG) characterized by persistent inflammation and salivary dysbiosis [27,28,29]. 

## 2. Materials and Methods

### 2.1. Animal Care, Ethics and Experimental Procedures

All experiments using rhesus macaques were approved by the Tulane Institutional Animal Care and Use Committee (Protocol No-3781) The Tulane National Primate Research Center (TNPRC) is an Association for Assessment and Accreditation of Laboratory Animal Care International accredited facility (AAALAC #000594). The NIH Office of Laboratory Animal Welfare assurance number for the TNPRC is A3071-01. All clinical procedures, including administration of anesthesia and analgesics, were carried out under the direction of a laboratory animal veterinarian. Animals were anesthetized with ketamine hydrochloride for blood collection procedures. Animals were pre-anesthetized with ketamine hydrochloride, acepromazine and glycopyrrolate, intubated and maintained on a mixture of isoflurane and oxygen. All possible measures were taken to minimize the discomfort of all the animals used in this study. Tulane University complies with NIH policy on animal welfare, the Animal Welfare Act and all other applicable federal, state and local laws.

### 2.2. Animal Model and Experimental Design

Nineteen age- and weight-matched male Indian rhesus macaques divided into four groups (Table 1) were used for these studies. Out of the eight, Group 1 vehicle untreated chronically SIV-infected (VEH-untreated/SIV) rhesus macaques, seven each were used for global microRNA expression (all but IJ60) and RNA-seq (all but P673) studies. For immunofluorescence studies, six age- and weight-matched male Indian rhesus macaques (Table 1) were randomly assigned to two groups. One group (*n* = 3; Group 2) received twice daily injections of vehicle (VEH/SIV) (1:1:18 of emulphor:alcohol:saline) and the other group (*n* = 3; Group 3) received twice daily injections of Δ^9^-THC (THC/SIV) beginning four weeks prior to SIV infection until 6 months post SIV infection. Five uninfected control (Group 4) age- and weight-matched male Indian rhesus macaques (Table 1) were used for global microRNA expression and RNA-seq studies. The absence of vehicle treatment in Group 1 rhesus macaques is unlikely to impact the studies as we recently demonstrated that vehicle treatment alone did not make any differences to the outcome [25]. In that study, rhesus macaques that did (IH96, HV48, IN24 and JC81) and did not receive (FT11, GH25, HB31, GA19 and HD08) vehicle showed no differences in *DEFA4* and *DEFA6* gene expression after SIV infection [25] and therefore were categorized as VEH/SIV RMs. In contrast, *DEFA4* and *DEFA6* expression in colon of THC treated SIV-infected macaques did not differ from uninfected control macaques. Chronic administration of VEH (Group 2) or Δ^9^-THC (Group 3) was initiated four weeks before SIV infection at 0.18 mg/kg as used in previous studies [24,25,26,30,31]. This dose of Δ^9^-THC was found to eliminate responding in a complex operant behavioral task in almost all animals [31]. All macaques with the exception of JJ71 were infected intravenously with 100TCID_50_ dose of the CCR5 tropic SIVmac251. Macaque JJ71 was infected with the molecular clone SIVmac239. The dose was subsequently increased for each subject to 0.32 mg/kg, over a period of approximately two weeks when responding was no longer affected by 0.18 mg/kg on a daily basis (i.e., tolerance developed), and maintained for the duration of the study. The optimization of the THC dosing in rhesus macaques accounts for the development of tolerance during the initial period of administration. Because in previously published studies [30,31] this dose of THC showed protection, the same dose was used in this study. The 0.32 mg/kg dose was also shown to be effective in SIV-infected rhesus macaques of Chinese origin [32]. SIV levels in plasma and OPM were quantified using the TaqMan One-Step Real-time RT-qPCR assay that targeted the LTR gene [24,25]. At necropsy, OPM tissue segments were collected in RNAlater (Thermo Fisher Scientific) and Z-fix for total RNA extraction and embedding in paraffin blocks. 

### 2.3. Global MicroRNA (miRNA) Profiling

Micro-RNA expression profiling was performed using TaqMan OpenArray Human MicroRNA panels (Thermo Fisher Scientific) [24,25,33,34] and RNA-seq (Novogene Inc, Sacramento, CA, USA), respectively. Briefly, total RNA from OPM tissue was isolated using the miRNeasy total RNA isolation kit (Qiagen Inc., Germantown, MD USA) following the manufacturer’s protocol. Approximately 100 ng total RNA were first reverse transcribed using the micro-RNA reverse transcription reaction kit (Thermo Fisher Scientific, Austin, TX, USA). 

Briefly, two master mixes representing either OpenArray panel (Panels A and B) were prepared for each RNA sample, which consisted of the following reaction components: 0.75 μL MegaPlex RT primers (10×), 0.15 μL dNTPs with dTTP (100 mM), 1.50 μL MultiScribe™ Reverse Transcriptase (50 U/μL), 0.75 μL 10× RT Buffer, 0.90 μL MgCl_2_ (25 mM), 0.09 μL RNase Inhibitor and 0.35 μL nuclease-free water (20 U/μL). Three microliters of total RNA (100 ng) were loaded into appropriate wells of a 96-well plate together with 4.5 μL of the RT reaction master mix. After a brief spin and 5 min of incubation on ice, samples in the 96-well plate were subjected to the following thermal cycling conditions on the ABI 7900 HT Fast PCR system: standard or max ramp speed, 16 °C for 2 min, 42 °C for 1 min, 50 °C 1 s (40 cycles), 85 °C 5 min, 23 °C. Immediately after thermal cycling, the 96-well plate containing cDNA was stored at −80 °C.

For pre-amplification, 2.5 μL of the cDNA from each sample were mixed with a total of 22.5 μL of pre-amplification reaction master mix consisting of 12.5 μL TaqMan^®^ PreAmp Master Mix (2×), 2.5 μL Megaplex™ PreAmp Primers (10×) and 7.5 μL nuclease-free water in a 96-well plate. After a brief vortex and spin, samples in the 96-well plate were subjected to the following thermal cycling conditions on the ABI 7900 HT Fast PCR system: standard or max ramp speed, hold 95 °C 10 min; hold 55 °C 2 min; hold 72 °C 2 min; 12 cycles at 95 °C 15 s and 60 °C 4 min; hold 4 °C. The preamplified product was diluted 40 times by mixing 4 μL of the preamplified product with 156 μL of 0.1× TE pH 8.0 and loaded onto TaqMan^®^ OpenArray^®^ human micro-RNA plates for processing using the QuanStudio™ 12K Flex Real-Time PCR system (Life Technologies). 

### 2.4. RNA-Seq Library Construction, Clustering and Sequencing

Transcriptome profiling by RNA-seq and data analysis were performed by Novogene Inc. cDNA library construction and sequencing were performed by Novogene Co. Ltd., Beijing, CA (http://www.novogene.cn/). For library construction, ~3 μg of total RNA from each sample were used to enrich mRNA with the poly-T oligo-attached magnetic beads. The purified mRNA was then randomly cleaved into small fragments using NEBNEXT RNA fragmentation buffer (NEB, Ipswich, MA, USA) following the manufacturer’s instructions. First-strand cDNA was synthesized using random hexamers and M-MuLV Reverse Transcriptase (RNase H-). Second-strand cDNA synthesis by nick translation was subsequently performed using *E. coli* DNA polymerase I and RNase H. Remaining overhangs were converted into blunt ends via exonuclease/polymerase activities. The final cDNA library was prepared after a round of purification, terminal repair, A-tailing, ligation of sequencing adapters, size selection and PCR enrichment. The cDNA fragments of preferentially 250−300 bp in length were selected using with AMPure XP system (Beckman Coulter, Irving, TX, USA.) and PCR amplified using Phusion High-Fidelity DNA polymerase (NEB, Ipswich, MA, USA), Universal PCR primers and Index (X) Primer. PCR products were then purified using AMPure XP system (Beckman Coulter, Irving, TX, USA), and library quality was assessed on the Agilent Bioanalyzer 2100 system (Agilent Technologies, Cedar Creek, TX, USA.). The clustering of the index-coded samples was performed on a cBot Cluster Generation System, using TruSeq SR Cluster Kit v3-cBot-HS (Illumina, San Diego, CA, USA), according to the manufacturer’s instructions. After cluster generation, the library preparations were sequenced on an Illumina Novaseq 6000 platform and 150 bp paired end reads were generated.

### 2.5. Cloning of 3′-UTR of AGR2 and TSC22D3 mRNA and Dual-Glo Luciferase Reporter Gene Assay

The 3′ UTR of the rhesus macaque *AGR2* and *TSC22D3* mRNA contains a single predicted miR-29b binding site (TargetScan 7.2) [24,25,33,34]. Accordingly, two separate short 34 nucleotide sequences containing the predicted miR-29b site on the 3′ UTRs of *AGR2* (5′-UGAUUUCUUUGGCCCCUGGACUA**UGGUGCU**CUA-3′) and *TSC22D3* (5′-UUUGAAUGCCAAACCCACCAUUCA**UGCU**GAC-3′) were synthesized (IDTDNA Technologies Inc., Coralville, IA, USA) for cloning into the pmirGLO Dual-Luciferase vector (Promega Corp, Fitchburg, WI, USA). Two separate oligonucleotides with the miR-29b binding site deleted (*n* = 7 nucleotides) on the 3′ UTR sequence of *AGR2* (5′-UGAUUUCUUUGGCCCCUGGACUACUA-3′) and *TSC22D3* (5′-UUUGAAUGCCAAACCCACCAUUCAGAC-3′) were also synthesized to serve as a negative control. The oligonucleotide sequences were synthesized with a *Pme1* site on the 5′ and *Xba1* site on the 3′ end for directional cloning. The pmirGLO vector was first cut with *Pme1* and *Xba1* restriction enzymes, gel purified and ligated with either wild-type sequence containing the miR-29b binding site (*AGR2* or *TSC22D3*-wtUTR) or deleted sequence (*AGR2* or *TSC22D3*-delUTR). We used HEK293 cells for the reporter assay as >95–98% of these cells can be successfully transfected with exogenous nucleic acids using the Lipojet^®^ transfection reagent (Signagen, Gaithersburg, MD, USA). HEK293 cells were plated at a density of 2 × 10^4^ cells per well of a 96-well plate. At 60–70% confluence, cells were co-transfected with ~100 ng *AGR2*, *TSC22D3*-wtUTR, *AGR2* or *TSC22D3*-delUTR luciferase reporter vector and 20 and 40 nM miR-29b or negative control (cel-miR-67-3p) mimic using the Lipojet^®^ transfection reagent (Signagen). After 72 h, the Dual Glo luciferase assay was performed according to the manufacturer’s recommended protocol (Promega Corp,) using the BioTek H4 Synergy plate reader (BioTek, Winooski, VT). The normalized *firefly* to *renilla* ratio was calculated to determine the relative reporter activity. Experiments were performed in 6 replicates and repeated thrice. 

### 2.6. Immunofluorescence for Cellular Localization of AGR2, WFDC2 and TSC22D3 in OPM Tissues

Immunofluorescence studies for the detection of *AGR2* (1 in 100 dilution) (Abcam, Cat No: ab227584), *WFDC2* (1 in 250 dilution) (Abcam, Cat No: ab200828) and *TSC22D3* (1 in 50 dilution) (Abcam, Cat No: ab197987) were performed as described previously [24,33,34]. Epithelial expression of *AGR2*, *WFDC2* and *TSC22D3* positive cells was confirmed using cytokeratin (1 in 500) (Biocare, Denmark) and appropriate Alexa fluorophor conjugated secondary antibodies (Thermo Fisher Scientific, Austin, TX, USA). Localization of *AGR2* protein expression to the endoplasmic reticulum (ER) was confirmed using the ER marker GRP94 (1 in 1000 dilution) (Abcam, Cat No: ab210960).

### 2.7. Quantitative Image Analysis of OPM Sections

Briefly, two slides containing OPM sections from each animal were stained with antibodies specific for all three marker genes. No differences in staining intensity were detected between slides for each macaque. For quantification, images from ten fields per slide were collected for each of the five macaques shown in Figures 2–4. Quantitation of cells and regions of interest [35] labeled by *AGR2, WFDC2* and *TSC22D3* was performed using Volocity 5.5 software (PerkinElmer, Austin, TX, USA) after capturing images on a Leica confocal microscope. Several ROI were hand drawn on the epithelial, lamina propria and submucosal regions containing MiSGs and their excretory ducts in the images captured from OPMs of VEH or THC treated chronically SIV-infected rhesus macaques (Groups 2 and 3 in Table 1) and processed utilizing the same brightness, density and black level settings. The data were graphed using Prism v8 software (GraphPad software, San Diego, CA, USA). 

### 2.8. miR-29b Overexpression Studies

To determine the impact of miR-29b on *TSC22D3*, we overexpressed FAM labeled locked nucleic [36] conjugated miR-29b mimics (Qiagen Inc.) in A549 (ATCC^®^ CCL-85) adenocarcinomic human alveolar basal epithelial cell line) cell line as these cells abundantly expressed *TSC22D3* protein. Initial screening of TR147 oral carcinoma and A253 submaxillary salivary epithelial cells revealed complete lack of *TSC22D3* protein expression in both cell types. A549 cells were cultured at a density of 5 × 10^5^ cells in each well of an eight-well chamber slide (Thermo Fisher Scientific) in complete DMEM containing 10% FBS and antibiotics and antimycotics. At 90% confluence, cells were transfected with two different concentrations of FAM-LNA-miR-29b or FAM-LNA-negative control mimic using the Lipojet transfection reagent (Signagen). Cells were fixed with 2% paraformaldehyde at 96 h post transfection and immunostained with *TSC22D3* and later with DAPI for nuclear localization. 

### 2.9. Quantitation of Mucosal Viral Loads

Total RNA samples from all SIV-infected animals were subjected to a quantitative real-time TaqMan One-step RT-qPCR analysis to determine the viral load in plasma and colon tissue. Briefly, primers and probes specific to the SIV LTR sequence were designed and used in the real-time TaqMan PCR assay. Probes were conjugated with a fluorescent reporter dye (FAM) at the 5′ end and a quencher dye at the 3′ end. Fluorescence signal was detected with an ABI Prism 7900 HT sequence detector (Thermo Fisher Scientific). Data were captured and analyzed with Sequence Detector Software Thermo Fisher Scientific). Viral copy number was determined by plotting *C*_T_ values obtained from the colon and jejunum samples against a standard curve (*y* = −3.36*x* + 38.6) (*r*^2^ = 0.999) generated with in vitro transcribed RNA representing known viral copy numbers. 

### 2.10. Data Analysis and Availability

For RNA-seq analysis, raw reads were first processed through in-house perl scripts to remove reads containing adapter or ploy-N or with base quality score lower than 20. At the same time, the Q20, Q30 and GC contents of the clean data were calculated. The clean reads were aligned to the genome assembly of *Macaca mulatta* 10 (https://www.ncbi.nlm.nih.gov/genome/215?genome_assembly_id=468623) using TopHat2, [37] and read numbers mapped to each gene were calculated using the HTSeq program [38]. The fragments per kilobase of transcript sequence per million base pairs of each gene were determined by the length of the gene and read counts mapped to this gene. Differential expression analyses of VEH- or THC-treated SIV-infected rhesus macaques and control groups were performed using the DESeq of R package [39]. Genes with a *p*-value <0.05 and |log2 fold change| > 1.5 were defined as differentially expressed.

QuantStudio™ run files from all groups were imported into ExpressionSuite software v1.0.2 (Thermo Fisher Scientific) and simultaneously analyzed using the uninfected control group as the calibrator to obtain relative gene expression values. MiRNA expression data were normalized to a combination of three endogenous controls RNU44, RNU48 and snoU6. In all experiments, the C_T_ upper limit was set to 28. A p-value of less than or equal to 0.05 (≤0.05) was considered significant. OpenArray TaqMan miRNA and Novogene RNA-seq data have been deposited with GEO and can be accessed using the following superseries record link (https://www.ncbi.nlm.nih.gov/geo/query/acc.cgi?acc=GSE151298).

*Firefly/Renilla* ratios were statistically analyzed using one-way ANOVA and post hoc multiple testing using Benjamini–Hochberg method.

## 3. Results

### 3.1. Plasma Viral Loads, CD4^+^ and CD8^+^ T Cell Status and Oral Histopathology

All SIV-infected rhesus macaques (Groups 1–3) had a wide range (0.02 × 10^6^ to 300 × 10^6^/mg RNA) of viral RNA copies in the OPM at ~180 days post infection (Table 1). We did not have plasma and OPM viral loads for IJ60. However, the read counts for several interferon stimulated genes, chemokines and anti-viral genes such as *MX1* were ~3–3.5-fold higher than uninfected controls. The high *MX1* expression indirectly suggested active viral replication. Longitudinal peripheral blood viral loads, CD4^+^ and CD8^+^ T cell dynamics from the three VEH/SIV (JH47, JR36, JD66) and THC/SIV (JI45, JT80, IV90) rhesus macaques were recently published [25]. Marked depletion of CD4^+^ T cells in the intestine and peripheral blood was detected in all six animals [25]. CD4^+^ T cell depletion was accompanied by a concomitant increase in CD8^+^ T cell percentages [25]. No differences in plasma and OPM viral RNA copies were detected between these two groups [25]. Although plasma viral RNA copy numbers did not differ between the three VEH/SIV (JH47, JR36 and JD66) and three THC/SIV (JI45, JT80 and IV90) macaques (Table 1), we detected higher numbers of SIV gp120 (KK41 antibody) positive CD3^+^ T cells in OPM tissue in 3/3 VEH/SIV (JH47, JR36 and JD66) but only 1/3 THC/SIV (JI457, JT80 and IV90) rhesus macaques (Figure S1). The detection of reduced numbers of SIVgp120/CD3^++^ in THC/SIV rhesus macaques is interesting but needs further verification using larger OPM sections and in a larger number of THC treated SIV-infected rhesus macaques.

### 3.2. Genes Associated with Anti-Viral Defense, Interferon Signaling and Toll-Like Receptor Signaling Are Markedly Upregulated in OPM of VEH/SIV but not THC/SIV Rhesus Macaques

**Figure 1:**
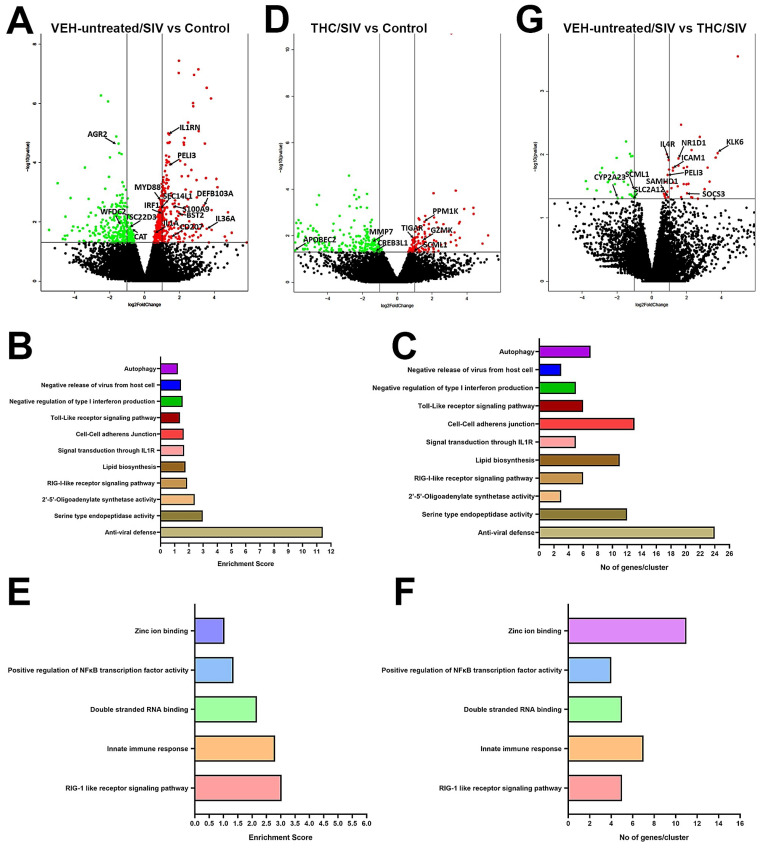
Changes in mRNA expression in the whole OPM tissue of chronically VEH-untreated or delta-9-tetrahydrocannabinol (∆^9^-THC) treated SIV-infected rhesus macaques. Volcano plot shows the relationship between fold-change (*X*-axis) and statistical significance (*Y*-axis) of differentially expressed mRNAs in VEH-untreated/SIV (**A**) and THC/SIV (**D**) rhesus macaques relative to controls and in VEH-untreated/SIV relative to THC/SIV rhesus macaques (**G**). The vertical lines in (**A**,**D**,**G**) correspond to 2.0-fold up and down, respectively, and the horizontal line represents *p* ≤ 0.05. The negative log of statistical significance (*p*-value) (base 10) is plotted on the *Y*-axis, and the log of the fold change base (base 2) is plotted on the *X*-axis. Notable differentially expressed mRNAs are listed in the volcano plots. DAVID functional annotation cluster analysis of upregulated genes in VEH-untreated/SIV (**B**,**C**) and THC/SIV rhesus macaques. (**E**,**F**). Top clusters of genes upregulated in VEH-untreated/SIV (**B**) and THC/SIV (**E**) rhesus macaques. Number of genes represented in each cluster in VEH-untreated/SIV (**C**) and THC/SIV (**F**) rhesus macaques.

To gain deeper insights into the molecular pathogenesis of HIV/SIV induced oral mucosal dysfunction and its modulation by THC, we performed RNA-seq of OPM samples collected at necropsy. Relative to uninfected controls, 461 genes were found to be significantly upregulated and differentially expressed in OPM of VEH-untreated/SIV rhesus macaques. Out of these, we successfully annotated 362 genes using DAVID (Figure 1A). Further, gene enrichment analysis using DAVID (NIAID) showed differential enrichment (Figure 1B,C) of biological functions involved in anti-viral defense (*n* = 24) (*p* = 1.3 × 10^18^), serine type endopeptidase activity (*n* = 12) (*p* = 7.0 × 10^−3^), 2′-5′-Oligoadenylate synthetase activity (*n* = 3) (*p* = 1.93 × 10^−3^), RIG-I-like receptor signaling pathway (*n* = 6) (*p* = 1.0 × 10^−2^), lipid biosynthesis (*n* = 11) (*p* = 2.8 × 10^−4^), signal transduction through IL1R (*n* = 5) (*p* = 3.0 × 10^−3^), cell–cell adherens junction (*n* = 13) (*p* = 1.2 × 10^−2^), negative regulation of type I interferon production (*n* = 5) (*p* = 2.0 × 10^−3^), negative release of virus from host cell (*n* = 3) (*p* = 3.3 × 10^−2^), Toll-like receptor signaling pathway (*n* = 6) (*p* = 5.0 × 10^−2^) and autophagy (*n* = 7) (*p* = 3.4 × 10^−2^). Table S1 lists the Top 50 differentially upregulated genes in OPM of VEH-untreated SIV rhesus macaques. Notable genes that were significantly upregulated exclusively in OPM of VEH-untreated/SIV rhesus macaques (Figure 1A) included *IL1RN* (inhibits activity of interleukin-1) [40], *PELI3* (interacts with complex containing *IRAK* kinases and *TRAF6* of *IL1* and *TLR* signaling pathways) [41], *BST2* (Interferon induced anti-viral protein) [42], alarmins (*IL1A*, *IL36A* and *S100A9*) [43,44], *IRF1* (innate and adaptive immune response) [45], *DEFB103A* (antimicrobial peptide), *CD207* (major receptor on langerhans cells for *Candida* species) [46], *STAT2* (type 1 interferon signaling) [45] and *MYD88* (adapter protein in Toll-like receptor signaling) [47]. 

In contrast, fewer genes (*n* = 132) were significantly upregulated in OPM of THC/SIV rhesus macaques (Figure 1B) compared to uninfected controls. Out of these, 83 genes were annotated. This represented a 3.5-fold reduction (362 vs. 83) in the total number of upregulated genes in THC/SIV compared to VEH-untreated/SIV rhesus macaques. Gene enrichment analysis using DAVID (NIAID) showed differential enrichment (Figure 1E,F) of biological functions involved in RIG-1 like receptor signaling pathway (*n* = 5) (*p* = 8.0 × 10^−5^), innate immune response (*n* = 7) (*p* = 8.7 × 10^−5^), double stranded RNA binding (*n* = 5) (*p* = 1.2 × 10^−4^), positive regulation of NFκB transcription factor activity (*n* = 4) (*p* = 7.0 × 10^−3^) and zinc ion binding (*n* = 12) (*p* = 1.4 × 10^−2^). While some of these functional annotation clusters overlapped with VEH-untreated/SIV rhesus macaques (Figure 1B,C), it is important to note that fewer genes associated with proinflammatory pathways comprised the clusters detected in THC/SIV rhesus macaques demonstrating the potent anti-inflammatory effects of long-term cannabinoid administration in the oral cavity in untreated HIV/SIV infection. We do not think that the fewer upregulated genes in OPM tissue of THC/SIV rhesus macaques is due to the small sample size (*n* = 3) because even with five animals in our previous study focused on the intestine [25] we detected a similar anti-inflammatory effect of THC. Moreover, similar to our study, several recently published studies have used sample sizes of three rhesus or cynomolgus macaques per group to investigate the pathogenesis of viral diseases (SARS-CoV-2, HIV) [48,49,50], asthma [51], LPS mediated inflammation [52] and tuberculosis [53]. A noteworthy gene is *MYD88*, a key signaling component of the Toll-like receptor signaling pathway cluster that was significantly upregulated in OPM of VEH-untreated/SIV (Figure 1A) but not THC/SIV rhesus macaques. Table S2 lists the Top 50 differentially upregulated genes in OPM of THC/SIV rhesus macaques. Genes of interest that were exclusively upregulated in OPM of THC/SIV rhesus macaques (Figure 1D) included *PPM1K* (regulates mitochondrial pore permeability and promotes cell survival) [54], *TIGAR* (protects against DNA damage induced apoptosis) [55], *SCML1* (maintain the transcriptionally repressive state of homeotic genes) [56] and *GZMK* (cytolysis of intracellular pathogens) [57]. 

### 3.3. Endoplasmic Reticulum Stress (ER) regulator Anterior Gradient 2 (AGR2), Epithelial Barrier Enhancing WAP Four-Disulfide Core Domain Protein 2 (WFDC2) and the Anti-Inflammatory TSC22D3 Are Significantly Downregulated in OPM of VEH-Untreated/SIV Rhesus Macaques

Compared to uninfected controls, 321 and 253 genes were downregulated in OPM of VEH-untreated/SIV and THC/SIV rhesus macaques, respectively. Out of these, 256 and 200 genes were successfully annotated in the VEH-untreated/SIV and THC/SIV groups, respectively. At least five genes with well-established functions in maintaining oral epithelial barrier integrity *AGR2* [58,59], *WFDC2* [60,61], anti-inflammatory function (*TSC22D3*) [62,63,64] and prevention of oxidative stress [48,65] were found to be significantly downregulated in OPM of VEH-untreated/SIV rhesus macaques (Figure 1D). On the other hand, proinflammatory extracellular matrix degrading proteinases (*MMP7*) [66], endoplasmic reticulum (ER) stress regulating and anti-viral *CREB3L1* [67] and DNA demethylating *APOBEC2* [68] were significantly downregulated in OPM of THC/SIV rhesus macaques (Figure 1D). Tables S3 and S4 lists the Top 50 differentially downregulated genes in OPM of VEH-untreated/SIV and THC/SIV rhesus macaques, respectively. The anti-inflammatory effects of THC became more distinctive when gene expression was compared between VEH-untreated/SIV and THC/SIV rhesus macaques (Figure 1G). Consistent with the presence of a persistent inflammatory state (Figure 1A and Figure S1I) in the OPM, VEH-untreated/SIV rhesus macaques showed significantly higher expression of *SAMHD1* (anti-viral restriction factor and mediation of TNFα proinflammatory responses) [69], *NR1D1* (transcription factor that controls macrophage and neutrophilic inflammation) [70], *PELI3* (transmission of TLR and IL-1 signaling via IRAK1/TRAF6 complex), *IL4R* (chemokine production and inflammation) [71], *KLK6* (inflammation and T cell activation) [72], *ICAM1* (lymphocyte trafficking and bacterial adherence) [73] and *SOCS3* (negative regulator of JAK-STAT signaling) [74] (Figure 1G). Tables S5 and S6 lists the Top 17 differentially up- and downregulated genes in OPM of VEH-untreated/SIV compared to THC/SIV rhesus macaques.

### 3.4. Chronic THC Administration Preserved AGR2, WFDC2 and TSC22D3 Protein Expression in Minor Salivary Glands (MiSGs) of Chronic SIV-Infected Rhesus Macaques

The interesting finding on the significant downregulation of *AGR2*, *WFDC2* and *TSC22D3* gene expression in OPM of VEH-untreated/SIV macaques (Table S3) further prompted us to investigate which cell types in the OPM contributed to its differential protein expression. More importantly, since the expression of all three genes was not decreased in OPM of THC/SIV rhesus macaques, we hypothesized that by inhibiting inflammation THC would preserve *AGR2*, *WFDC2* and *TSC22D3* protein expression. We focused on these three genes for the following reasons. First, *AGR2* is a protein disulfide isomerase family of endoplasmic reticulum proteins that catalyzes protein folding, trafficking and assembly of cysteine-rich transmembrane receptors and intestinal/salivary gland mucins [58,59]. In addition, *AGR2* also regulates cell migration, differentiation and adhesion [59]. Similarly, *WFDC2* is a matrix metalloproteinase inhibitor that was recently demonstrated to play a critical role in intestinal epithelial barrier function and preventing dysbiosis, bacterial invasion and translocation [60,61]. Further, *WFDC2* is expressed by MiSGs [60,61] and in association with other related *WAP* domain containing proteins functions in epithelial host defense. Finally, the expression of *TSC22D3*, also known as glucocorticoid induced leucine zipper (*GILZ*), is stimulated by glucocorticoids and *IL10* and is well described to play a key role in mediating the anti-inflammatory and immunosuppressive effects of both proteins [62,63,64]. 

**Figure 2:**
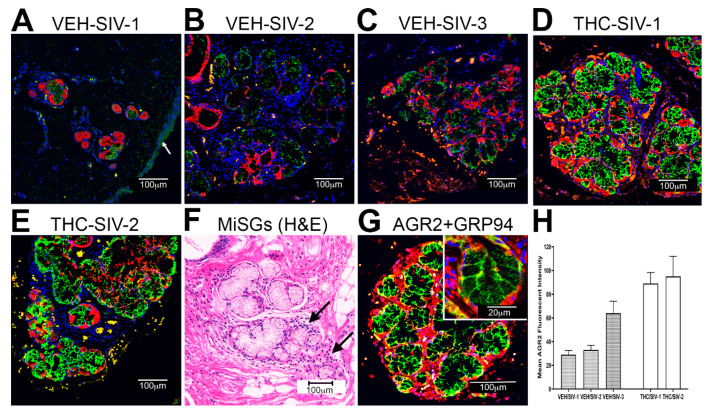
Chronic THC administration preserves *AGR2* protein expression in the minor salivary glands (MiSGs) of chronically SIV-infected rhesus macaques (**A**–**E**) Triple labels with *AGR2*, cytokeratin (**red**) and Topro3 (**blue**) for nuclear staining. Note the significantly decreased AGR2 (**A**–**C**) staining in the MiSGs of VEH/SIV rhesus macaques. In contrast, *AGR2* (**D**,**E**) staining is intense in the MiSGs of THC/SIV rhesus macaques. All panels are 40× magnification. An H&E image of OPM tissue from a VEH/SIV rhesus macaque (**F**) shows mild to moderate lymphoplasmacytic infiltration (black arrows) of MiSGs suggesting sialadenitis. (**G**) Triple labels with *AGR2*, endoplasmic reticulum marker *GRP94* (**red**) and Topro3 (**blue**) for nuclear staining. Yellow staining in (**G**) (inset) indicates localization of *AGR2* to the endoplasmic reticulum. Quantification of cells and regions of interest (ROI) labeled by *AGR2* (**H**) was performed using Volocity 5.5 software after capturing images on a Leica confocal microscope. Several ROI were hand drawn on the MiSGs and their ductal regions in the images from the three VEH/SIV and two THC/SIV rhesus macaques. The mean fluorescent intensity data are represented as mean ± standard deviation for each animal.

**Figure 3:**
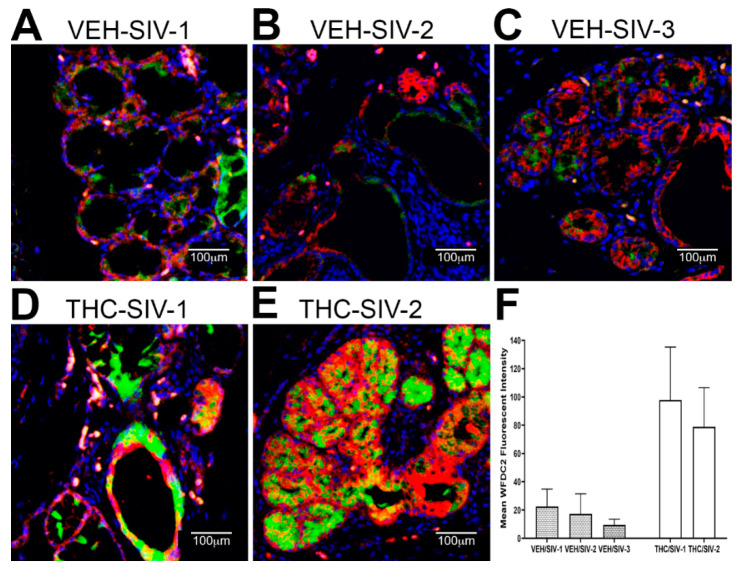
Chronic THC administration preserves *WFDC2* protein expression in the minor salivary glands (MiSGs) of chronically SIV-infected rhesus macaques. (**A**–**E**) Triple labels with *WFDC2* [48], cytokeratin (**red**) and Topro3 (**blue**) for nuclear staining. Note the significantly decreased *WFDC2* (**A**–**C**) staining in the MiSGs of the VEH/SIV rhesus macaques. In contrast, *WFDC2* (**D**,**E**) staining is intense in the MiSGs and salivary ducts of THC/SIV rhesus macaques. All panels are 40× magnification. Quantification of cells and regions of interest (ROI) labeled by *WFDC2* (**F**) was performed using Volocity 5.5 software after capturing images on a Leica confocal microscope. Several ROI were hand drawn on the MiSG regions in the images from three VEH/SIV and two THC/SIV rhesus macaques. The mean fluorescent intensity measurements for each animal are represented as mean ± standard deviation.

**Figure 4:**
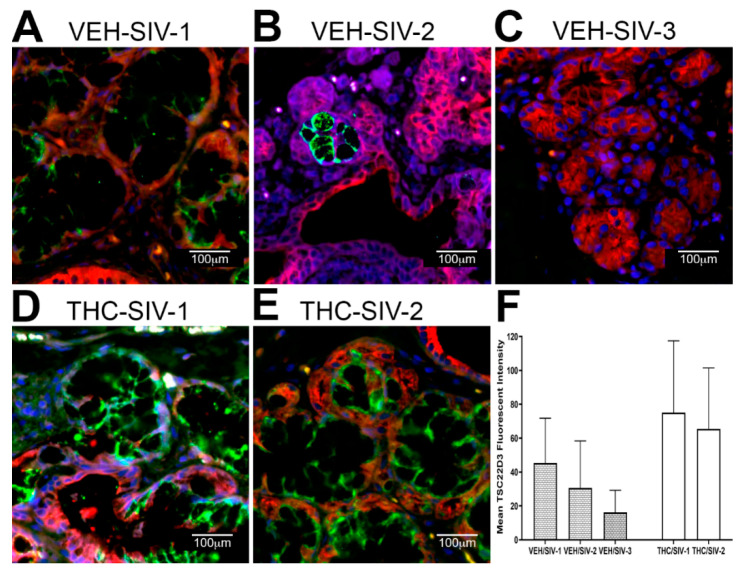
Chronic THC administration preserves *TSC22D3* protein expression in the minor salivary glands (MiSGs) of chronically SIV-infected rhesus macaques. (**A**–**E**) Triple labels with *TSC22D3* [48], cytokeratin (**red**) and Topro3 (**blue**) for nuclear staining. Note the significantly decreased *TSC22D3* (**A**–**C**) staining in the minor salivary glands of the VEH/SIV rhesus macaques. In contrast, *TSC22D3* (**D**,**E**) staining is intense in the MiSGs of the THC/SIV rhesus macaques. All panels are 40× magnification. Quantification of cells and regions of interest (ROI) labeled by *TSC22D3* (**F**) was performed using Volocity 5.5 software after capturing images on a Leica confocal microscope. Several ROI were hand drawn on the MiSG regions in the images from three VEH/SIV and two THC/SIV rhesus macaques. The mean fluorescent intensity measurements for each animal are represented as mean ± standard deviation.

For immunofluorescence analysis, we did not include untreated control animals (Group 4) as the mRNA read counts for all three genes (*AGR2, WFDC2* and *TSC22D3*) were significantly higher than that detected in VEH-untreated/SIV rhesus macaques (Table S3). Since none of the three genes were downregulated in the OPM of THC/SIV rhesus macaques, we decided to compare differences in protein expression between VEH/SIV (JH47, JR36 and JD66) and THC/SIV (JI45, JT80 and IV90) rhesus macaques. In agreement with RNA-seq data, *AGR2* staining intensity was considerably weaker in OPM of VEH/SIV (Figure 2A–C) compared to the THC/SIV rhesus macaques (Figure 2D,E), suggesting that chronic HIV/SIV infection is associated with marked downregulation of *AGR2* protein expression in the OPM. In contrast, *AGR2* staining intensity was brighter and stronger in the OPM of THC/SIV rhesus macaques (Figure 2D,E). Interestingly, based on cytokeratin expression (marker of epithelial cells), *AGR2* protein expression localized exclusively to the minor salivary gland (MiSGs) mucus secretory epithelial cells and its secretory ducts (lined by simple cuboidal epithelium) present in the submucosal layer of the OPM (Figure 2A–E). This is evident in Figure 2F showing a hematoxylin–eosin stained section of the OPM containing a small cluster of MiSGs with predominantly mucus secretory acini. Note the markedly stronger *AGR2* staining intensity in the MiSG clusters present in the OPM of THC/SIV (Figure 2D,E) compared to VEH/SIV (Figure 2A–C) rhesus macaques. Further, Figure 2F shows mild to moderate inflammatory cell (T cells and plasma cells) infiltration of the MiSGs in a VEH/SIV RM, suggesting the presence of MiSG sialadenitis. Compared to reports in the intestinal epithelium, very weak *AGR2* staining was detected in the stratified squamous epithelium lining the OPM (Figure 2A)). Since *AGR2* has been described to regulate ER-stress, we stained the sections with the ER marker, *GRP94*. Consistent with its function in maintaining ER homeostasis, *GRP94* confirmed subcellular localization of *AGR2* protein to the ER of MiSG acini (Figure 2G). The inset in Figure 2G clearly shows that the *AGR2* stained regions also stained with antibodies recognizing the ER protein *GRP94*, confirming the expression of *AGR2* within the ER of MiSG mucus secretory epithelial cells. Quantitative image analysis of all stained MiSG clusters confirmed elevated *AGR2* protein expression in OPM of THC/SIV relative to VEH/SIV rhesus macaques (Figure 2H). Unfortunately, the OPM sections collected in Z-fix for immunofluorescence studies from one of the THC/SIV (JT80) rhesus macaques (Table 1) did not contain MiSGs and, therefore, immunofluorescence images from only two THC/SIV rhesus macaques are shown in Figure 2H. Since at least three samples are required to generate a *p* value, we were unable to statistically analyze the between group (VEH/SIV vs. THC/SIV) differences in mean fluorescent intensity values shown in Figure 2H, as well as in Figure 3F and Figure 4F. 

The decreased mRNA expression of *WFDC2* and *TSC22D3* was equally intriguing as both proteins play important roles in inhibiting metalloprotease activity, mucosal inflammation and maintaining the intestinal epithelial barrier function and by extension the oral epithelial barrier integrity [60,61,62,63,64]. Similar to *AGR2*, protein expression of both *WFDC2* (Figure 3) and *TSC22D3* (Figure 4) localized exclusively to the MiSG acini and salivary ducts. Consistent with the mRNA data, expression of both proteins was markedly lower in OPM of VEH/SIV rhesus macaques (Figure 3A–C and Figure 4A–C). In contrast, protein expression of *WFDC2* and *TSC22D3* was considerably higher in THC/SIV rhesus macaques (Figure 3D–F and Figure 4D–F). *TSC22D3* protein expression was extremely low and barely detectable in one of the VEH/SIV RMs (Figure 4C) compared to the other two (Figure 4A,B). Digital image quantification using Volocity confirmed markedly enhanced expression of *WFDC2* and *TSC22D3* proteins in the MiSGs of THC/SIV rhesus macaques (Figure 3F and Figure 4F). Due to the small sample size, the data on the anti-inflammatory effects of THC should be interpreted with caution. Nevertheless, the findings provide new information on the pathogenesis of oral manifestations in HIV patients even on anti-retroviral therapy and have translational relevance for the mitigation of oral mucosal inflammation. Therefore, these translational findings need to be confirmed in a larger cohort of THC treated SIV-infected rhesus macaques.

### 3.5. TSC22D3 Is Post-Transcriptionally Regulated by miR-29b

**Figure 5:**
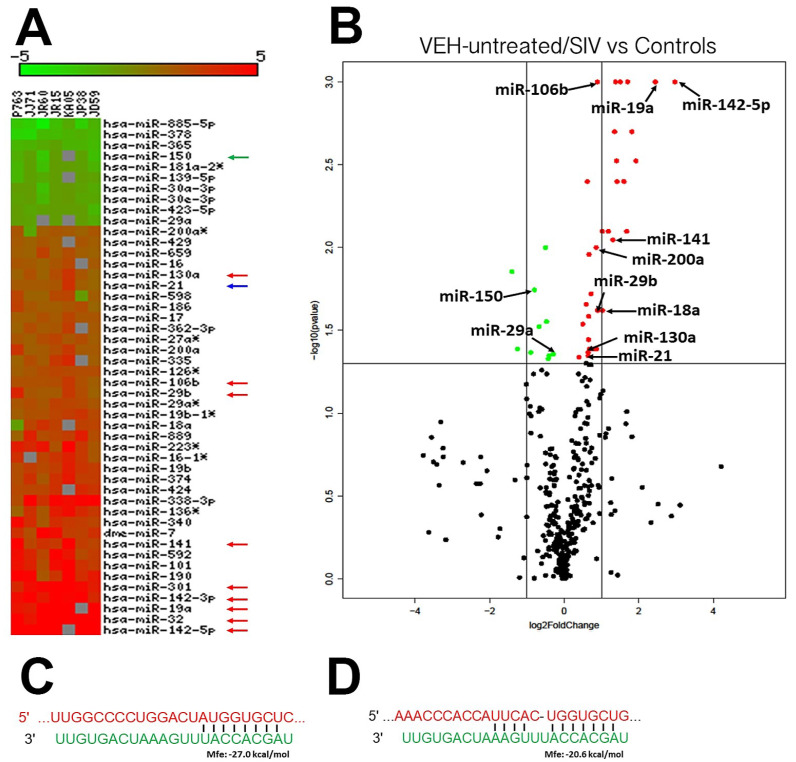
Changes in miRNA expression in the whole OPM tissue of chronically VEH-untreated SIV-infected rhesus macaques. The heat map shows all differentially expressed (*p* ≤ 0.05) miRNAs in the OPM of VEH-untreated/SIV vs. controls (**A**). miRNA species originating from the opposite arm of the precursor are denoted with an asterisk (*). Volcano plot shows the relationship between fold-change (*X*-axis) and statistical significance (*Y*-axis) of differentially expressed miRNAs in VEH-untreated/SIV rhesus macaques relative to controls (**B**). The vertical lines in (**B**) correspond to 2.0-fold up and down, respectively, and the horizontal line represents *p* ≤ 0.05. The negative log of statistical significance (*p*-value) (base 10) is plotted on the *Y*-axis, and the log of the fold change base (base 2) is plotted on the *X*-axis in (**B**). Green and red arrows indicate, respectively, downregulated and upregulated miRNAs common to VEH-untreated/SIV rhesus macaques. The location of miRNAs of interest is denoted with arrows in (**B**). miRNA–mRNA duplex showing a single miR-29b binding site (seed nucleotide region) on the rhesus macaque *AGR2* (**C**) and *TSC22D3* (**D**) mRNA 3′ untranslated region.

To determine the post transcriptional mechanisms regulating gene expression, we profiled miRNA expression in the same OPM (Figure 5A,B) samples from VEH-untreated/SIV rhesus macaques (*n* = 7) and identified 48 (38 upregulated and 10 downregulated) differentially expressed (DE) miRNAs relative to uninfected controls (*n* = 5). In terms of magnitude, miR-19a, miR-301, miR-142-3p, miR-32 and miR-142-5p were among a select list of miRNAs that showed the highest upregulation in OPM (bottom of heat map in Figure 5A and black arrows in volcano plot in Figure 5B). An important finding is the significant upregulation in OPM (Figure 5A,B) of miR-21 (blue arrow in Figure 5A and volcano plot in Figure 5B), a miRNA known to regulate periodontitis [75], T-cell activation [76] and oral carcinoma [77]. Interestingly, RNA-seq for gene expression profiling also confirmed miR-21 upregulation in OPM of VEH-untreated/SIV rhesus macaques. Further, expression of miR-150 was significantly downregulated (green arrow in Figure 5A and volcano plot in Figure 5B), a miRNA we previously demonstrated to be downregulated during T cell activation in the intestine [34]. Unfortunately, due to limitations in RNA availability, we could not perform miRNA profiling in the OPM of THC/SIV rhesus macaques. However, our previously published studies showed that THC suppressed proinflammatory miR-21 and miR-222 expression in the intestine [25]. Additionally, our unpublished studies in the brain of chronic SIV-infected rhesus macaques identified a similar suppressive effect of THC on proinflammatory miRNA expression. Therefore, we expect THC to suppress proinflammatory miRNA expression in the OPM. 

**Figure 6:**
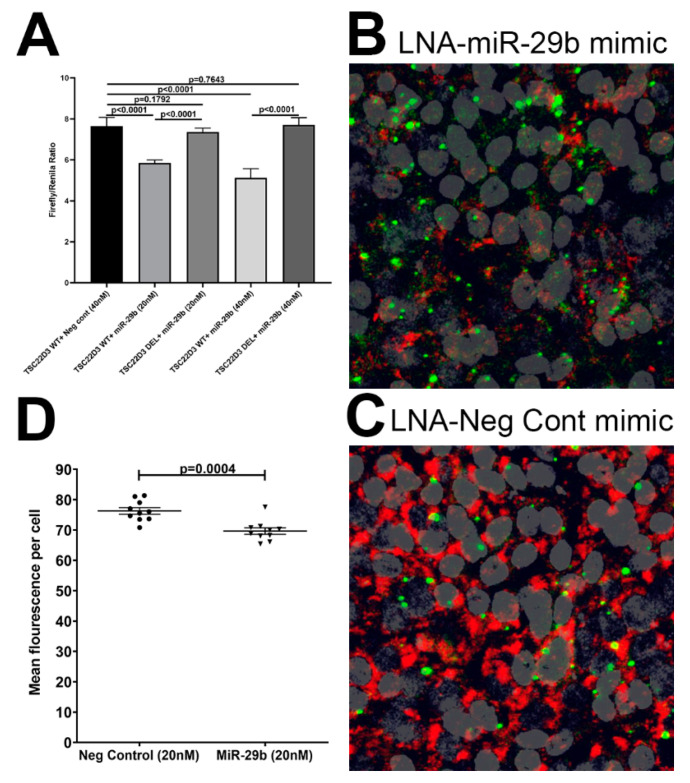
*TSC22D3* is a direct target of miR-29b. Luciferase reporter vectors containing a single highly conserved miR-29b (**A**) binding site (seed nucleotide region) on the rhesus macaque *TSC22D3* mRNA 3′ UTR or the corresponding construct with the binding sites deleted were co-transfected into HEK293 cells with 20 or 40 nM miR-29b or Negative control mimic. *Firefly* and *Renilla* luciferase activities were detected using the Dual-Glo luciferase assay system 96 h after transfection. Note significantly reduced *Firefly*/*Renilla* ratios following co-transfection of miR-29b mimic with a pmirGLO vector containing wild-type (WT) miRNA binding sites (**A**). Luciferase reporter assays were performed thrice in six replicate wells. Representative immunofluorescence images (**B**,**C**) and quantification (**D**) showing reduction in *TSC22D3* protein expression 96 h post transfection of A549 cells in triplicate wells with LNA-conjugated FAM labeled miR-29b (**B**) or negative control (**C**) mimics. miR-29b overexpression followed by immunofluorescence detection of *TSC22D3* protein was performed twice in triplicate wells. *Firefly/Renilla* ratios were analyzed using one-way ANOVA followed by Tukey’s post hoc test. Western blot densitometry data were analyzed using unpaired “*t*” test. A *p* value of ≤0.05 was considered significant.

To identify potential molecular mechanisms regulating *AGR2* and *TSC22D3* expression in the OPM, we performed a bioinformatic analysis using the TargetScan 7.2 algorithm [78] to identify potential miRNAs upregulated in the OPM that are predicted to directly regulate *AGR2, WFDC2* and *TSC22D3*. The bioinformatic analysis identified perfect miRNA seed nucleotide matches (miRNA nucleotide positions 2–7) for miR-29b on the 3′ mRNA UTR of *AGR2* (Figure 5C) (conserved in human, chimp and rhesus macaque) and *TSC22D3* (Figure 5D) (conserved in up to thirteen different mammalian species) that had a minimum free energy of ≤27.0 and 20.6 kcal/mol, respectively. Moreover, miR-29 dysregulation has been confirmed in other pathological conditions (tumors) of the salivary gland [79]. As shown in Figure 6A, transfection of HEK293 cells with both 20 and 40 nM LNA-conjugated miR-29b mimic significantly reduced firefly/renila ratios suggesting that miR-29b can regulate *TSC22D3* expression by directly binding to its 3′ UTR and exerting posttranscriptional repression. Although miR-29b binding to the *AGR2* 3′ UTR decreased firefly/renila ratios, the reduction detected only with 20 nM miR-29b mimic was very minimal (10%) (Figure S2) compared to that detected with *TSC22D3* (Figure 6A). Next, we overexpressed FAM-labeled LNA-conjugated miR-29b mimics in A549 cells to determine its effect on *TSC22D3* protein expression. We used A549 cells as these cells abundantly expressed *TSC22D3* protein. Two other cells lines, namely TR147 (oral carcinoma cell line) and A253 (submaxillary salivary gland cell line), were also evaluated for overexpression studies. Unfortunately, neither cell line expressed the *TSC22D3* protein. We preferred the current approach over other detection methods such as Western blotting as it enabled the quantification of *TSC22D3* protein [25] solely in cells that were successfully transfected with the miR-29b mimic (green fluorescing cells). This approach also ensured that *TSC22D3* protein expression was not quantified in miR-29b untransfected cells. Figure 6B clearly shows that miR-29b overexpression effectively decreased *TSC22D3* protein expression in A549 cells compared to cells that were transfected with the negative control mimic (Figure 6C). Moreover, target downregulation was successfully achieved with a low physiological dose (20 nM) of miR-29b mimic (Figure 6D). Overall, these data provide a potential miRNA mediated post-transcriptional mechanism regulating MiSG inflammation in chronic HIV/SIV infection. 

## 4. Discussion

Despite viral suppression by cART, chronic oral inflammation, thought to be a key driver of oral mucosal dysfunction, does not fully subside to baseline levels [1,2,3,4,5,6,10]. This can contribute to breakdown of the oral epithelial barrier resulting in oral microbial and by-product translocation, systemic immune activation and accelerated disease progression [7]. Here, we investigated the impact of long-term administration of the naturally occurring phytocannabinoid in the *Cannabis sativa* plant, namely THC, and demonstrate its anti-inflammatory effects in the oral cavity of cART naïve chronically SIV-infected rhesus macaques.

Given the paucity of information on the molecular mechanisms underlying HIV/SIV-induced oral mucosal dysfunction, we first profiled mRNA expression in OPM tissues of chronic SIV-infected rhesus macaques. Consistent with inflammatory signaling emanating from increased viral replication, we found enrichment of biological functions associated with antiviral defense (*n* = 24 genes), type I interferon signaling pathway (*n* = 18 genes), innate immunity (*n* = 30 genes), positive regulation of interferon-alpha (*n* = 4) and beta production (*n* = 3), signal transduction through IL1R (*n* = 5 genes) and Toll-like receptor signaling pathway (*n* = 6 genes). These gene expression signatures suggest a host response reflective of both viral replication and concurrent bacterial dysbiosis in the oral cavity. This is further supported by the identification of a molecular profile of “alarmin” gene expression that is consistent with microbial dysbiosis. This included upregulation of the antimicrobial peptide *DEFB103* (10.5-fold), *IL36* (11.9-fold), *IL1A* (1.7-fold) and *S100A9* (2.4-fold). Similar to our previous studies in the intestine [25], we detected an approximately 3.4-fold reduction in the number of upregulated transcripts in OPM of THC/SIV compared to control macaques. In contrast, chronic THC treatment selectively upregulated the expression of *TIGAR, SCML1* and *GZMK*. Overall, the marked reduction in the number of significantly upregulated proinflammatory genes in the OPM of THC/SIV macaques suggests that cannabinoids exert strong anti-inflammatory effects in the oral mucosa.

Another important finding was the significant downregulation of *AGR2, WFDC2, TSC22D3* and *CAT* mRNA in OPM of VEH-untreated/SIV macaques relative to uninfected control macaques. *AGR2* is a protein disulfide isomerase that functions to facilitate protein folding in the endoplasmic reticulum (ER). Deletion of *AGR2* in mice resulted in ER stress in the intestinal epithelium, disrupted paneth cell homeostasis, and the animals developed Crohn’s disease-like granulomatous ileocolitis [58]. Additionally, *AGR2* was demonstrated to play a critical role in the normal maturation and secretion of *MUC2* mucin in murine colonic goblet cells [59]. *WFDC2* is a metalloproteinase inhibitor that was recently shown to protect the intestinal epithelial barrier and at the same time prevent dysbiosis, microbial invasion and translocation [61]. Finally, *TSC22D3*, also known as glucocorticoid-induced leucine zipper (*GILZ*), is a glucocorticoid and IL-10 induced protein that exerts its anti-inflammatory effects by inhibiting the *NFκB* and *MAPK* pathways [63]. Interestingly, reduced expression of *TSC22D3* is associated with adipose, vascular and pulmonary inflammation [80]. On the contrary, overexpression of *TSC22D3* successfully protected mice against a lethal form of septic peritonitis [81]. *CAT* is a key antioxidant enzyme that protects against oxidative stress by converting reactive oxygen species such as hydrogen peroxide to water and oxygen. Taken together, these interesting findings on the relatively reduced expression of key anti-inflammatory proteins may help sustain chronic oral inflammation in HIV/SIV infection and that THC may inhibit inflammation by preserving their expression in the OPM. 

We next performed immunofluorescence analysis to determine if changes in *AGR2, WFDC2* and *TSC22D3* gene expression were reflected at the protein level. Interestingly, all three proteins localized exclusively to the minor salivary glands (MiSGs) and/or their secretory ducts in the OPM with protein expression being markedly higher in the THC/SIV rhesus macaques. The oral cavity contains about 600–1000 capsule-free MiSGs that measure ~1–5 mM in diameter [82]. They are distributed throughout the oral mucosa, palate, lips and tongue [82] and located in the submucosa, surrounded by connective tissue, or embedded between muscle fibers [82]. Most MiSGs contain mucus acini and secrete mucus saliva, which creates a protective lubricating micron-thick film that helps to avoid the subjective feeling of dry mouth [82,83]. Additionally, MiSGs produce saliva during sleep and therefore a decreased MiSG flow rate due to HIV/SIV induced inflammation may partially explain the night time dry mouth reported by HIV patients [84,85,86]. Although MiSGs contribute less than 10% of the saliva volume [82,83], they secrete high concentrations of immunoglobulin A that protects the oral mucosa from bacterial invasion [82,83]. Therefore, HIV/SIV induced inflammation of the MiSGs may reduce secretory IgA production and consequently impair oral innate immune defenses. Such an environment can drive major shifts in the relative proportions of beneficial and harmful bacteria eventually leading to dysbiosis. 

To identify a potential mechanism regulating gene expression, we profiled miRNA expression in the OPM of VEH-untreated/SIV rhesus macaques. Several classical inflammation-associated miRNAs; miR-21, miR-106b, miR-142-3p, miR-142-5p, miR-17, miR-186 and miR-29b were significantly upregulated in OPM of VEH-untreated/SIV macaques. The most notable miRNA was miR-21 that has been previously linked to T cell activation [76] and, more importantly, oral squamous cell carcinoma [77]. miR-21 was also detected by RNA-seq done to identify differential gene expression, thus confirming its upregulation by two independent techniques. Similarly, miR-142-3p has functional associations with immune activation and oral squamous cell carcinoma [87]. In agreement with studies reported by us and others, we detected decreased expression of miR-150, a miRNA known to be downmodulated during T and B cell activation [34]. Bioinformatic analysis identified miR-29b to have predicted binding sites on the 3′ mRNA UTR of *AGR2* and *TSC22D3*. Luciferase reporter assays and miRNA overexpression studies confirmed the ability of miR-29b to post-transcriptionally regulate *TSC22D3*. Due to limited RNA availability, we did not perform miRNA profiling in OPM of THC/SIV rhesus macaques. However, our recently published studies in the intestine [25] and unpublished studies in the brain have confirmed that chronic THC treatment abrogated the expression of inflammation associated miRNAs. Therefore, these data collectively suggest that oral mucosal dysfunction is characterized by widespread dysregulation of miRNA expression and that miR-29b, in particular, can potentially bind and post transcriptionally downregulate *TSC22D3* protein expression.

Dysregulated miRNAs have been shown to perturb immune responses and stimulate proinflammatory cytokine and autoantibody production, all of which contribute to the pathogenesis of autoimmune diseases such as Sjogren’s syndrome (SS) that affect primarily the salivary and lacrimal glands [88,89]. In addition, affected individuals experience both xerostomia and sialadenitis, the latter characterized by inflammatory cell infiltration of the major and minor salivary glands that in due course results in oral dysbiosis. Currently, there is no cure and the available treatments for this syndrome are symptomatic. While it has been suggested that cannabis and cannabinoids could potentially alleviate some of the symptoms associated with SS and other autoimmune diseases, studies describing their long-term efficacy and safety are still lacking. The findings from our current and previously published [25] rhesus macaque studies provide evidence that long-term low-dose cannabinoids can exert immune-modulatory effects without any observed adverse effects (psychotropic effects and xerostomia), and they can improve the overall quality of life of people living with HIV and potentially those affected by autoimmune diseases such as SS.

In summary, the present study describes genome wide changes in gene expression and its modulation by cannabinoids in the OPM in chronic HIV/SIV infection. Additionally, our findings demonstrate a significant impact of HIV/SIV infection on the MiSGs in the OPM and the potential for miR-29b to post-transcriptionally downregulate *TSC22D3* protein expression in MiSG acini. These changes may explain the high incidence of dry mouth (xerostomia) experienced by HIV-infected patients [84,85]. More importantly, the low THC dose (0.32 mg/kg) administered parenterally in the present study successfully prevented HIV/SIV induced inflammatory signaling in the MiSGs without causing any adverse psychotropic effects or xerostomia. Although preliminary, these protective effects of THC in the oral cavity are very intriguing and more detailed studies to further confirm these findings including mechanisms are needed in the future. Since we also detected a gene expression signature consistent with dysbiosis, future studies are needed to determine if cannabinoids inhibit oral dysbiosis. Finally, human clinical trials using low-dose orally-administered cannabinoids in combination with anti-retroviral drugs to reduce oral mucosal inflammation and systemic chronic immune activation/inflammation need to be conducted in the future.

## Figures and Tables

**Table 1:** Vehicle or Tetrahydrocannabinol (∆^9^-THC) treated chronic SIV-infected and uninfected rhesus macaques.

Animal ID	SIV Inoculum	Duration of Infection	Plasma Viral Loads 10^6^/mL	OPM Viral Loads 10^6^/mg RNA	Opportunistic Infections
**Chronic SIV-Infected and Vehicle Untreated (Group 1) for microRNA and gene expression studies**					
P763 #	SIVmac251	145	NA	3	ND
JJ71 # *	SIVmac239	148	NA	0.3	ND
JR60 # *	SIVmac251	180	NA	10	ND
JR15 # *	SIVmac251	180	NA	0.3	ND
KA05 # *	SIVmac251	90	NA	0.7	ND
JP38 # *	SIVmac251	180	NA	0.1	ND
JD59 # *	SIVmac251	150	NA	300	ND
IJ60 *	SIVmac251	180	NA	NA	ND
**Chronic SIV-Infected and Vehicle treated (Group 2) for Immunofluorescence studies**					
JH47 %	SIVmac251	180	2	NA	ND
JR36 %	SIVmac251	180	0.5	0.02	ND
JD66 %	SIVmac251	180	0.04	0.5	ND
**Chronic SIV-Infected and ∆^9^-THC treated (Group 3) for Immunofluorescence studies**					
JI45 # % *	SIVmac251	180	3	0.02	ND
JT80 # % *	SIVmac251	180	1	9	ND
IV90 # % *	SIVmac251	180	0.02	0.3	ND
**Uninfected Controls (Group 4) for microRNA and gene expression studies**					
JC65 # *	NA	NA	NA	NA	NA
GJ01 # *	NA	NA	NA	NA	NA
GK11 # *	NA	NA	NA	NA	NA
GK22 # *	NA	NA	NA	NA	NA
JD95 # *	NA	NA	NA	NA	NA

NA, not available, ND, none detected; # denotes animals used for microRNA expression studies; * denotes animals used for gene expression (RNA-seq) studies; % denotes animals used for *AGR2*, *WFDC2* and *TSC22D3* immunofluorescence studies.

## Supplementary Materials

The following are available online at https://www.mdpi.com/1999-4915/12/7/713/s1, Figure S1. SIV positive cells are considerably higher in OPM of VEH/SIV rhesus macaques: (A–C) triple labels with SIV gp120 (KK41 clone), CD3 and macrophage (CD163 in green). Note the relatively higher numbers of KK41/CD3 positive cells in VEH/SIV rhesus macaques (A–C) in the OPM lamina propria. In contrast, fewer SIV positive (KK41) CD3^+^ T cells were detected in the OPM of THC/SIV rhesus macaques (D,E). All panels are 40× magnification. Mild green (CD163) background staining was detected in the stratified squamous epithelium lining the OPM (B–H) and the salivary ducts (I). (G) A magnified image of a lymphoid follicle (boxed area in (c)) present in the OPM lamina propria of VEH/SIV-3 rhesus macaque. A magnified image of the OPM lamina propria of THC/SIV-3 (boxed area in (F)) shows fewer SIV positive (KK41) CD3^+^ T cells (H). Note the periductal KK41/CD3^++^ T cell infiltration (red arrow) of salivary gland in VEH/SIV-3 (I) confirming periductular sialadenitis. White arrow points to the large salivary duct. Figure S2. miR-29b may post transcriptionally regulate *AGR2* expression. Luciferase reporter vectors containing a single highly conserved miR-29b binding site (seed nucleotide region) on the rhesus macaque *AGR2* mRNA 3′ UTR or the corresponding construct with the binding sites deleted were co-transfected into HEK293 cells with 20 or 40 nM miR-29b or Negative control mimic. *Firefly* and *Renilla* luciferase activities were detected using the Dual-Glo luciferase assay system 96 h after transfection. Note a small (10%) but statistically significant reduction in *Firefly*/*Renilla* ratios following co-transfection of 20 nM but not 40 nM miR-29b mimic with a pmirGLO vector containing wild-type (WT) miRNA binding sites. Luciferase reporter assays were performed thrice in six replicate wells.
