# Supplementary material for: Long Term Delta-9-tetrahydrocannabinol Administration Inhibits Proinflammatory Responses in Minor Salivary Glands of Chronically Simian Immunodeficieny Virus Infected Rhesus Macaques
Source: Viruses. 2020 Jul 1;12(7):713. doi: 10.3390/v12070713 (PMC7412369; doi:10.3390/v12070713)
Supplement: Supplementary file 1 [file viruses-12-00713-s001.zip › Supplemental Tables 1-6.pdf]

Supplemental Table 1. List of Upregulated genes in OPM of VEH-untreated/SIV compared to control rhesus macaques

Gene Symbol	Gene Name	Control Read Count	VEH-untreated/SIV Read Count	Fold Change	P value
HLA-DQB1	MHC Class II Antigen HLA-DQ-Beta-1	1.0	273.4	272.9	2.12E-02
KLK6	Kallikrein Related Peptidase 6	51.4	934.0	18.2	6.79E-04
IFI44	Interferon Induced Protein 44	80.8	1405.4	17.4	3.56E-04
DDX60	DExD/H-Box Helicase 60	245.8	2922.5	11.9	2.97E-07
IL36A	Interleukin 36 alpha	322.4	3826.9	11.9	1.70E-02
IFI6	Interferon Alpha Inducible Protein 6	429.0	4935.3	11.5	3.33E-04
IFI44L	Interferon Induced Protein 44 Like	21.5	236.0	11.0	2.27E-05
DEFB103	Defensin-beta 103	67.8	712.1	10.5	2.81E-03
KLK9	Kallikrein Related Peptidase 9	45.9	446.1	9.7	1.22E-02
IFIT3	Interferon Induced Protein with Tetratricopeptide Repeats 3	114.9	988.7	8.6	8.73E-06
ISG15	Interferon-Stimulated Protein, 15 KDa	547.6	4382.2	8.0	1.50E-03
MX1	Myxovirus Resistance Protein 1	360.8	2588.3	7.2	1.09E-07
IFIT1	Interferon Induced Protein with Tetratricopeptide Repeats 1	82.4	421.9	5.1	8.17E-04
OAS2	2'-5'-oligoadenylate	133.6	657.4	4.9	1.47E-05
KLK8	Kallikrein Related Peptidase 8	403.0	1959.8	4.9	4.75E-03
DDX58	DExD/H-Box Helicase 58	163.8	779.2	4.8	2.10E-05
DHX58	DExH-Box Helicase 58	114.6	449.8	3.9	3.65E-08
BST2	Bone Marrow Stromal Antigen 2	462.7	1810.0	3.9	2.54E-03
OAS1	2'-5'-Oligoadenylate Synthetase 1	291.2	1133.8	3.9	9.38E-08
GBP2	Guanylate Binding Protein 2	449.1	1744.2	3.9	4.81E-03
ERO1A	Endoplasmic Reticulum Oxidoreductase 1 Alpha	3634.5	13291.6	3.7	4.41E-03
APOL2	Apolipoprotein L2	255.8	880.6	3.4	2.49E-02
EPSTI1	Epithelial Stromal Interaction 1	53.5	171.9	3.2	1.15E-02
BNIP3	BCL2 Interacting Protein 3	399.7	1224.1	3.1	2.49E-03
KLK7	Kallikrein Related Peptidase 7	259.0	766.4	3.0	1.73E-02
SLC26A9	Solute Carrier Family 26 Member 9	895.0	2597.3	2.9	1.08E-03
CD207	Langerhans Cell Specific C-Type Lectin	173.5	501.9	2.9	3.54E-02
IFIT5	Interferon Induced Protein with Tetratricopeptide Repeats 5	57.3	164.0	2.9	5.41E-03
OASL	2'-5'-Oligoadenylate Synthetase Like	55.1	156.3	2.8	9.78E-03
IFIH1	Interferon Induced with Helicase C Domain 1	280.6	762.9	2.7	2.05E-05
STAT1	Signal Transducer and Activator of Transcription 1	2335.1	6225.8	2.7	1.13E-05
MAMU-A3	major histocompatibility complex, class I, A	855.6	2262.5	2.6	3.13E-02
RNF213	Ring Finger Protein 213	1041.5	2750.7	2.6	6.36E-05
PTPRH	Protein Tyrosine Phosphatase Receptor Type H	502.5	1324.2	2.6	4.05E-02
PELI3	Pellino E3 Ubiquitin Protein Ligase Family Member 3	94.9	249.0	2.6	9.47E-05
DDX60L	DEAD-Box Helicase 60 Like	48.5	125.9	2.6	3.86E-04
CLDN17	Claudin 17	537.0	1338.3	2.5	4.81E-02
RBP1	Retinol binding protein 1	182.0	448.9	2.5	3.30E-03
GAL9	Galectin 9	267.8	654.1	2.4	3.82E-04
B2M	beta-2-microglobulin	10609.1	25778.0	2.4	4.88E-04
PARP14	Poly (ADP-Ribose) Polymerase Family Member 14	237.5	573.3	2.4	3.54E-04
S100A9	S100 Calcium Binding Protein A9	99397.7	239638.3	2.4	3.19E-03
KRT78	Keratin 78	20592.8	49484.0	2.4	2.68E-04
SAMD9L	Sterile Alpha Motif Domain Containing 9 Like	352.5	827.7	2.3	5.51E-03
YOD1	YOD1 Deubiquitinase	199.6	454.9	2.3	1.06E-03
COL1A1	Collagen Type I Alpha 1 Chain	337.1	759.8	2.3	4.72E-02
TGFA	Transforming growth factor alpha	178.3	401.0	2.2	7.47E-04
KIFC3	Kinesin Family Member C3	694.3	1545.5	2.2	3.87E-03
LPIN1	Lipin 1	651.0	1428.1	2.2	1.44E-02
TRIM22	Tripartite Motif Containing 22	759.9	1664.4	2.2	7.57E-03

Supplemental Table 2. List of Upregulated genes in OPM of THC/SIV compared to control rhesus macaques

Gene Symbol	Gene Name	Control Read Count	THC/SIV Read Count	Fold Change	P value
IFI27	Interferon Alpha Inducible Protein 27	269.4	8048.0	29.9	2.19E-02
IFI44	Interferon Induced Protein 44	83.5	1743.7	20.9	1.15E-03
IFI44L	Interferon Induced Protein 44 Like	22.2	459.8	20.7	6.28E-04
DDX60	DExD/H-Box Helicase 60	254.0	3647.3	14.4	7.14E-04
ISG15	ISG15 Ubiquitin Like Modifier	565.4	6834.0	12.1	1.25E-02
IFI6	Interferon Alpha Inducible Protein 6	443.6	5353.9	12.1	2.55E-03
HERC6	HECT Domain and RCC1-Like Domain-Containing Protein 6	106.7	1247.5	11.7	2.97E-03
IFIT3	Interferon Induced Protein with Tetratricopeptide Repeats 3	118.7	1214.6	10.2	1.58E-02
XAF1	XIAP Associated Factor 1	23.8	184.0	7.7	6.47E-03
MX1	Myxoma Resistance Protein 1	372.7	2563.3	6.9	2.19E-02
DDX58	DExD/H-Box Helicase 58	169.3	983.6	5.8	6.76E-03
IFIT1	Interferon Induced Protein with Tetratricopeptide Repeats 1	85.2	487.4	5.7	2.48E-02
RSAD2	Radical S-Adenosyl Methionine Domain Containing 2	51.3	260.2	5.1	4.47E-03
IRF7	Interferon Regulatory Factor 7	187.9	886.1	4.7	1.77E-02
CMPK2	Cytidine/Uridine Monophosphate Kinase 2	41.8	180.9	4.3	1.60E-02
OAS1	2'-5'-Oligoadenylate Synthetase 1	300.8	1063.7	3.5	3.16E-03
DHX58	DExH-Box Helicase 58	118.4	413.8	3.5	5.04E-03
GZMK	Granzyme K	59.1	166.0	2.8	1.70E-02
TRIM22	Tripartite Motif Containing 22	786.1	2204.6	2.8	2.23E-02
IFIH1	Interferon Induced with Helicase C Domain 1	289.9	800.6	2.8	1.76E-02
DDX60L	DExD/H-Box 60 Like	50.2	123.4	2.5	4.54E-03
STAT1	Signal Transducer And Activator Of Transcription 1	2412.0	5690.6	2.4	2.14E-02
PPM1K	Protein Phosphatase, Mg2+/Mn2+ Dependent 1K	299.8	701.3	2.3	1.81E-03
TRIM14	Tripartite Motif Containing 14	275.5	640.8	2.3	4.28E-03
GBP2	Guanylate Binding Protein 2	464.1	1042.7	2.2	1.15E-02
LGALS9A	Galectin 9	276.7	620.6	2.2	5.60E-03
TRIM5	Tripartite Motif Containing 5	331.1	712.0	2.2	3.42E-02
PHTF2	Putative Homeodomain Transcription Factor 2	399.4	854.5	2.1	1.17E-02
HELZ2	Helicase with Zinc Finger 2	264.6	560.4	2.1	3.74E-02
SLFN5	Schlafen Family Member 5	599.8	1229.7	2.1	1.77E-02
PARP12	Poly (ADP-Ribose) Polymerase Family Member 12	258.1	528.2	2.0	6.81E-03
SCML1	Scm Polycomb Group Protein Like 1	116.5	236.0	2.0	4.61E-02
PARP14	Poly (ADP-Ribose) Polymerase Family Member 14	245.4	495.4	2.0	4.37E-02
KSR1	Kinase Suppressor of Ras 1	638.8	1264.6	2.0	3.59E-02
ADM	Adrenomedullin	105.8	208.7	2.0	1.96E-02
CHPT1	Choline Phosphotransferase 1	4771.3	9390.5	2.0	2.91E-02
LRRC17	Leucine Rich Repeat Containing 17	98.5	190.4	1.9	1.67E-02
B2M	Beta-2-Microglobulin	10966.5	21159.0	1.9	1.26E-02
CEP85	Centrosomal Protein 85	355.1	679.3	1.9	4.77E-02
TIGAR	TP53 Induced Glycolysis Regulatory Phosphatase	366.2	695.6	1.9	1.46E-02
SAMD9L	Sterile Alpha Motif Domain Containing 9 Like	364.1	689.4	1.9	4.46E-02
APOL2	Apolipoprotein L2	264.5	499.1	1.9	1.58E-02
RNF213	Ring Finger Protein 213	1076.5	2024.7	1.9	2.10E-02
PHACTR2	Phosphatase And Actin Regulator 2	817.9	1536.1	1.9	1.85E-02
ABHD18	Abhydrolase Domain Containing 18	118.5	220.2	1.9	3.10E-02
RND3	Rho Family GTPase 3	4102.4	7479.3	1.8	1.53E-02
ARIH1	Ariadne RBR E3 Ubiquitin Protein Ligase 1	615.3	1101.4	1.8	4.89E-02
SNX24	Sorting Nexin 24	444.1	777.2	1.8	3.93E-02
UBL3	Ubiquitin Like 3	836.9	1454.2	1.7	4.68E-02
ESPL1	Extra Spindle Pole Bodies Like 1, Separase	249.1	430.8	1.7	4.03E-02

Supplemental Table 3. List of downregulated genes in OPM of VEH-untreated/SIV compared to control rhesus macaques

Gene Symbol	Gene Name	Control Read Count	VEH-untreated/SIV Read Count	Fold Change	P value
CHST4	Carbohydrate Sulfotransferase 4	245.6	80.1	-3.1	1.31E-05
SLC44A4	Solute Carrier Family 44 Member 4	308.7	102.4	-3.0	4.73E-03
AGR2	Anterior gradient 2, Protein Disulphide Isomerase Family Member	2465.5	880.7	-2.8	2.27E-05
AOX1	Aldehyde Oxidase 1	308.8	110.5	-2.8	2.49E-02
ABCC9	ATP Binding Cassette Subfamily C Member 9	109.8	40.7	-2.7	4.57E-03
NKX3-1	NK3 Homeobox 1	493.4	188.6	-2.6	2.56E-02
C4BPA	Complement Component 4 Binding Protein Alpha	249.0	95.9	-2.6	1.12E-02
TMC5	Transmembrane Channel Like 5	378.8	149.8	-2.5	4.80E-03
SEL1L3	SEL1L Family Member 3	545.3	217.5	-2.5	4.67E-02
S100A1	S100 Calcium Binding Protein A1	676.5	272.3	-2.5	5.18E-05
ABCA4	ATP Binding Cassette Subfamily A Member 4	137.6	57.4	-2.4	1.10E-02
WFDC2	WAP Four-Disulfide Core Domain 2	1377.6	583.7	-2.4	1.33E-02
FOLR1	Folate Receptor 1	225.7	99.3	-2.3	6.35E-04
TRPV4	Transient Receptor Potential Cation Channel Subfamily V Member 4	120.2	53.0	-2.3	1.53E-02
GOLM1	Golgi Membrane Protein 1	1255.0	571.0	-2.2	3.01E-03
MEF2C	Myocyte Enhancer Factor 2C	494.7	228.1	-2.2	4.86E-02
ARHGAP24	Rho GTPase Activating Protein 24	137.6	63.9	-2.2	2.33E-03
ZNF177	Zinc Finger Protein 177	156.3	72.8	-2.1	8.10E-03
CP	Ceruloplasmin	181.8	85.4	-2.1	3.18E-02
SYBU	Syntabulin	224.9	106.4	-2.1	1.22E-02
CDH11	Cadherin 11	263.2	125.6	-2.1	8.29E-04
TENT5C	Terminal Nucleotidyltransferase 5C	254.4	125.5	-2.0	4.77E-02
CREB3L4	CAMP Responsive Element Binding Protein 3 Like 4	140.2	70.0	-2.0	3.17E-03
KIAA1324	Estrogen-Induced Gene 121 Protein	552.5	277.8	-2.0	4.52E-02
KCNMA1	Potassium Calcium-Activated Channel Subfamily M Alpha 1	387.1	198.1	-2.0	1.41E-02
CMAH	CMP-N-Acetylneuraminic Acid Hydroxylase	373.5	191.2	-2.0	1.60E-03
UCHL1	Ubiquitin C-Terminal Hydrolase L1	145.2	74.5	-1.9	6.99E-03
PRR15L	Proline Rich 15 Like	170.9	88.7	-1.9	3.84E-02
CDCA7L	Cell Division Cycle Associated 7 Like	214.8	113.4	-1.9	3.04E-02
LRIG1	Leucine Rich Repeats and Immunoglobulin Like Domains 1	485.8	259.4	-1.9	5.86E-03
SERPINE2	Serpin Family E Member 2	306.0	164.6	-1.9	3.52E-02
TMEM45A	Transmembrane Protein 45A	174.3	95.0	-1.8	1.76E-02
ZFX	Zinc Finger Protein X-Linked	235.2	128.4	-1.8	6.76E-03
SLC22A17	Solute Carrier Family 22 Member 17	343.9	189.7	-1.8	6.41E-03
RGS16	Regulator of G Protein Signaling 16	215.7	122.2	-1.8	1.53E-02
ARSD	Arylsulfatase D	151.6	87.1	-1.7	3.55E-02
IQGAP2	IQ Motif Containing GTPase Activating Protein 2	310.6	180.9	-1.7	1.42E-02
MFSD4A	Major Facilitator Superfamily Domain Containing 4A	348.1	203.4	-1.7	2.70E-02
PDGFC	Platelet Derived Growth Factor C	192.1	113.3	-1.7	1.94E-02
ZNF608	Zinc Finger Protein 608	225.6	133.4	-1.7	1.12E-02
BCL6	B-Cell Lymphoma 6 Protein	910.7	540.5	-1.7	1.41E-02
FGF7	Fibroblast Growth Factor 7	161.0	96.3	-1.7	4.24E-02
FKBP5	FKBP Prolyl Isomerase 5	291.4	177.5	-1.6	3.26E-02
TSC22D3	Glucocorticoid-Induced Leucine Zipper Protein	1514.3	927.0	-1.6	2.09E-02
ANO1	Anoctamin 1	239.6	146.8	-1.6	2.28E-02
RNASE4	Ribonuclease A Family Member 4	2701.9	1667.4	-1.6	4.98E-02
MAGED1	MAGE Family Member D1	587.5	376.9	-1.6	4.57E-02
CAT	Catalase	981.2	636.0	-1.5	2.62E-02
CERK	Ceramide Kinase	604.8	392.1	-1.5	2.83E-02
PC	Pyruvate Carboxylase	297.4	194.4	-1.5	4.71E-02

Supplemental Table 4. List of downregulated genes in OPM of THC/SIV compared to control rhesus macaques

Gene Symbol	Gene Name	Control Read Count	THC/SIV Read Count	Fold Change	P value
NRAP	Nebulin Related Anchoring Protein	732.1	11.4	-64.3	1.86E-02
TPM1	Tropomyosin 1	1657.2	374.5	-4.4	2.15E-02
GOLM1	Golgi Membrane Protein 1	1296.6	490.4	-2.6	2.48E-02
HSPB7	Heat Shock Protein Family B (Small) Member 7	182.5	3.9	-46.7	2.88E-03
PFKM	Phosphofructokinase, Muscle	752.8	374.0	-2.0	3.42E-02
PGAM1	Phosphoglycerate mutase 1	305.3	13.1	-23.2	1.85E-02
GAMT	Guanidinoacetate N-Methyltransferase	180.9	91.1	-2.0	4.77E-02
MMP7	Matrix Metallopeptidase 7	154.7	52.5	-2.9	2.44E-02
TNNI2	Troponin I2, Fast Skeletal Type	1662.5	100.4	-16.6	4.39E-02
MLF1	Myeloid Leukemia Factor 1	125.0	19.1	-6.5	2.63E-02
MB	Myoglobin	3126.5	53.9	-58.0	1.98E-02
SYNM	Synemin	256.3	102.5	-2.5	4.96E-02
CP	Ceruloplasmin	187.6	56.7	-3.3	5.23E-03
RGS16	Regulator ff G Protein Signaling 16	222.9	81.1	-2.7	2.10E-03
SMARCD3	SWI/SNF Related, Matrix Associated, Actin Dependent Regulator of Chromatin, Subfamily D, Member 3	142.1	57.4	-2.5	1.38E-02
CKMT2	Creatine Kinase, Mitochondrial 2	194.8	8.5	-22.9	9.46E-03
CYB5R1	Cytochrome B5 Reductase 1	165.1	62.0	-2.7	1.88E-02
FHL1	Four and A Half LIM Domains 1	3395.6	820.3	-4.1	2.33E-02
NEB	Nebulin	1108.3	19.9	-55.7	2.83E-03
LMOD3	Leiomodin 3	167.7	3.3	-50.7	8.06E-03
SYNPO2	Synaptopodin 2	187.2	19.1	-9.8	3.74E-02
TTN	Titin	2823.0	64.3	-43.9	6.73E-03
CUEDC1	CUE Domain Containing 1	268.7	136.7	-2.0	3.90E-02
TRDN	Triadin	201.3	2.9	-70.4	7.92E-03
MYH8	Myosin Heavy Chain 8	321.3	12.1	-26.6	3.08E-02
MYH1	Myosin Heavy Chain 1	2232.5	2.0	-1099.6	2.83E-03
MYH2	Myosin Heavy Chain 2	4599.1	18.2	-252.1	5.50E-03
FOLR1	Folate Receptor Alpha	233.2	112.1	-2.1	3.39E-02
RERG	RAS Like Estrogen Regulated Growth Inhibitor	302.7	147.8	-2.0	3.28E-02
MYL1	Myosin Light Chain 1	1422.2	9.0	-157.3	4.25E-04
ENO3	Enolase 3	1609.9	31.1	-51.8	4.85E-03
XIRP2	Xin Actin Binding Repeat Containing 2	315.2	12.4	-25.4	6.35E-03
TMC5	Transmembrane Channel Like 5	391.5	117.9	-3.3	8.46E-03
CKM	Creatine Kinase, M-Type	2831.9	22.5	-126.0	4.61E-03
MYOZ1	Myozenin 1	868.4	8.1	-107.5	4.76E-03
LTF	Lactotransferrin	319.4	55.8	-5.7	3.94E-02
CYBA	Cytochrome B-245 Alpha Chain	456.1	244.4	-1.9	4.00E-02
NGFR	Nerve Growth Factor Receptor	215.5	106.8	-2.0	3.28E-02
PDLIM3	PDZ And LIM Domain 3	725.1	165.2	-4.4	3.10E-02
TPM2	Tropomyosin 2	2373.0	120.7	-19.7	2.15E-02
MYOT	Myotilin	366.0	5.3	-68.8	9.62E-03
RASSF4	Ras Association Domain Family Member 4	233.9	70.2	-3.3	6.35E-04
DES	Desmin	1423.1	44.9	-31.7	3.75E-03
CREB3L1	CAMP Responsive Element Binding Protein 3 Like 1	272.1	116.5	-2.3	4.22E-02
TGM2	Transglutaminase 2	211.0	59.2	-3.6	2.89E-02
SFRP1	Secreted Frizzled Related Protein 1	461.9	248.6	-1.9	3.76E-02
CSRP3	Cysteine And Glycine Rich Protein 3	200.9	2.2	-93.1	2.16E-02
ACTN2	Actinin Alpha 2	298.8	8.0	-37.1	2.66E-02
CMYA5	Cardiomyopathy Associated 5	675.1	36.8	-18.3	1.99E-02
APOBEC2	Apolipoprotein B MRNA Editing Enzyme Catalytic Subunit 2	382.0	4.6	-83.9	1.42E-02

Supplemental Table 5. List of Upregulated genes in OPM of VEH-untreated/SIV compared to THC/SIV rhesus macaques

Gene Symbol	Gene Name	VEH-untreated/SIV Read Count	THC/SIV Read Count	Fold Change	P value
RBP1	Retinol Binding protein 1	426.8	194.6	2.2	3.40E-02
SAMHD1	SAM Domain And HD Domain-Containing Protein 1	664.8	384.7	1.7	4.84E-02
PELI3	Pellino E3 Ubiquitin Protein Ligase Family Member 3	237.0	137.6	1.7	2.69E-02
IL4R	Interleukin 4 Receptor	951.9	479.6	2.0	1.73E-02
PAPSS2	3'-Phosphoadenosine 5'-Phosphosulfate Synthase 2	1471.9	804.6	1.8	9.87E-03
ICAM4	Intercellular Adhesion Molecule 4	38.9	13.8	2.8	3.19E-02
PXDC1	PX Domain-Containing Protein 1	237.7	146.5	1.6	4.15E-02
NSUN7	NOP2/Sun RNA Methyltransferase Family Member 7	225.5	120.2	1.9	2.10E-02
WFDC5	WAP Four-Disulfide Core Domain 5	116.4	17.2	6.8	5.27E-03
KLK6	Kallikrein Related Peptidase 6	891.2	64.6	13.8	9.54E-03
THEM4	Thioesterase Superfamily Member 4	143.2	85.7	1.7	4.41E-02
ICAM1	Intercellular Adhesion Molecule 1	259.0	109.8	2.4	2.79E-02
CD93	Complement Component C1q Receptor	90.6	39.2	2.3	1.79E-02
SLCO4A1	Solute Carrier Organic Anion Transporter Family Member 4A1	247.8	148.3	1.7	4.52E-02
SOCS3	Suppressor of cytokine signaling 3	50.3	12.6	4.0	2.95E-02
TMPRSS11F	Transmembrane Serine Protease 11F	426.8	218.0	2.0	1.22E-02
AGPAT2	1-Acylglycerol-3-Phosphate O-Acyltransferase 2	1450.1	829.6	1.7	4.16E-02

Supplemental Table 6. List of Downregulated genes in OPM of VEH-untreated/SIV compared to THC/SIV rhesus macaques

Gene Symbol	Gene Name	VEH-untreated/SIV Read Count	THC/SIV Read Count	Fold Change	P value
SYT8	Synaptotagmin 8	57.3	266.2	-4.6	1.93E-02
KATNAL1	Katanin Catalytic Subunit A1 Like 1	16.0	33.5	-2.1	4.67E-02
CFAP53	Cilia And Flagella Associated Protein 53	13.1	33.8	-2.6	3.01E-02
ZNF680	Zinc Finger Protein 680	35.8	70.4	-2.0	3.98E-02
ZNF837	Zinc Finger Protein 837	13.6	31.1	-2.3	4.28E-02
SNCAIP	Synuclein Alpha Interacting Protein	19.6	38.2	-2.0	4.79E-02
SCML1	Scm Polycomb Group Protein Like 1	117.9	217.9	-1.8	4.28E-02
PAPPA	Pappalysin 1	19.0	43.2	-2.3	1.80E-02
SPHKAP	SPHK1 Interactor, AKAP Domain Containing	27.3	75.9	-2.8	6.24E-03
MPP3	Membrane Palmitoylated Protein 3	17.6	42.1	-2.4	1.94E-02
ZNF177	Zinc Finger Protein 177	69.2	158.2	-2.3	1.07E-02
STYXL1	Serine/Threonine/Tyrosine Interacting Like 1	19.1	71.9	-3.8	1.18E-04
CYP2A23	Cytochrome P450 Family 2 Subfamily A Member 23	165.6	720.0	-4.3	2.94E-02
BAIAP3	BAI1 Associated Protein 3	37.7	81.4	-2.2	1.06E-02
SLC2A12	Solute Carrier Family 2 Member 12	54.8	110.7	-2.0	4.67E-02
NRBP2	Nuclear Receptor Binding Protein 2	23.9	45.7	-1.9	3.79E-02
RASSF10	Ras Association Domain Family Member 10	169.1	370.7	-2.2	4.52E-02
