# Supplementary figures and images for: Long Term Delta-9-tetrahydrocannabinol Administration Inhibits Proinflammatory Responses in Minor Salivary Glands of Chronically Simian Immunodeficieny Virus Infected Rhesus Macaques
Source: Viruses. 2020 Jul 1;12(7):713. doi: 10.3390/v12070713 (PMC7412369; doi:10.3390/v12070713)

**A** VEH-SIV-1

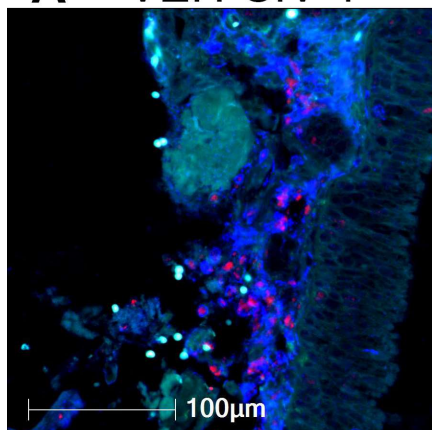

**B** VEH-SIV-2

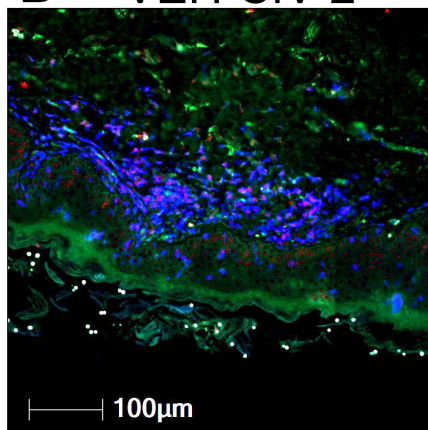

**C** VEH-SIV-3

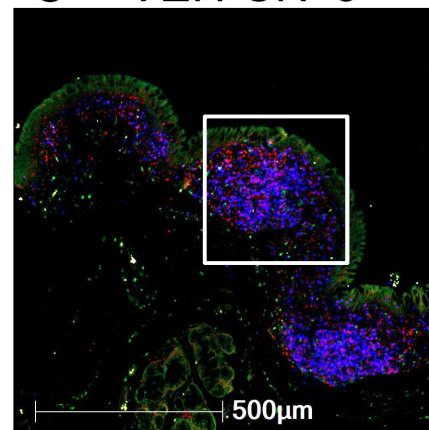

**D** THC-SIV-1

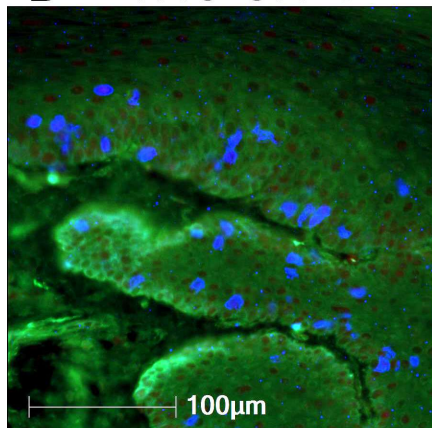

**E** THC-SIV-2

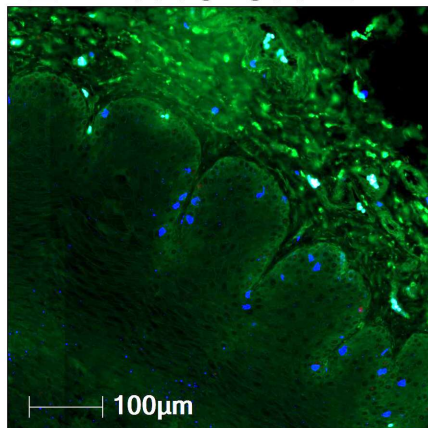

**F** THC-SIV-3

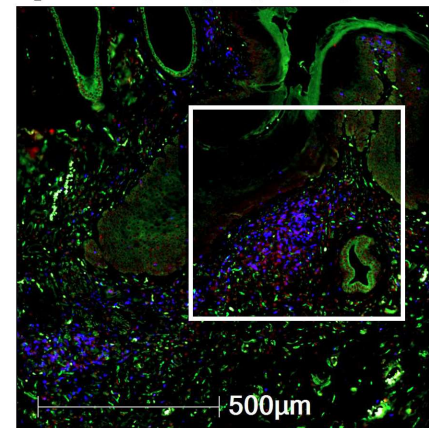

**G**

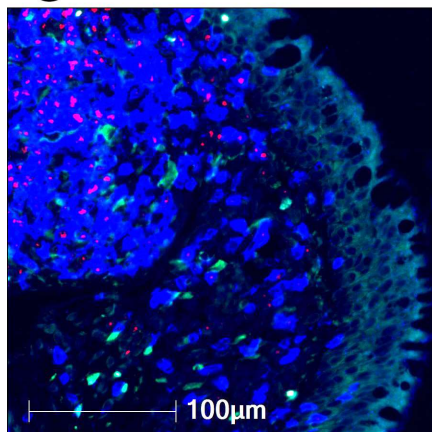

**H**

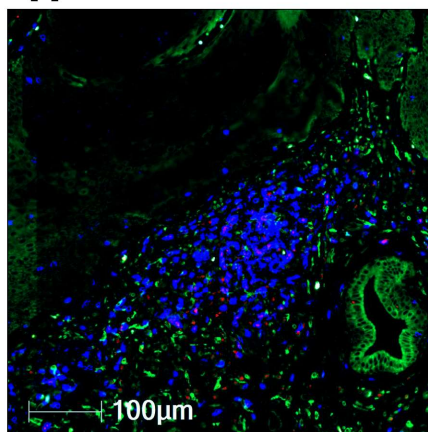

**I**

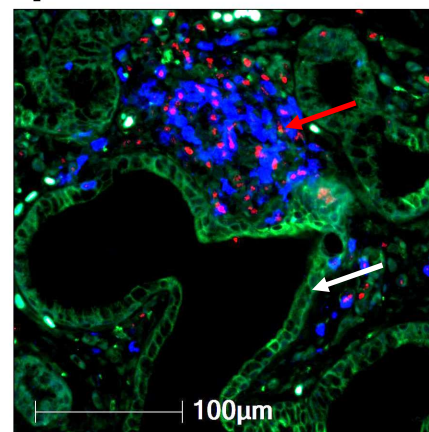

Supplement: Supplementary file 1 [file viruses-12-00713-s001.zip › Supplemental Figure S1.pdf]

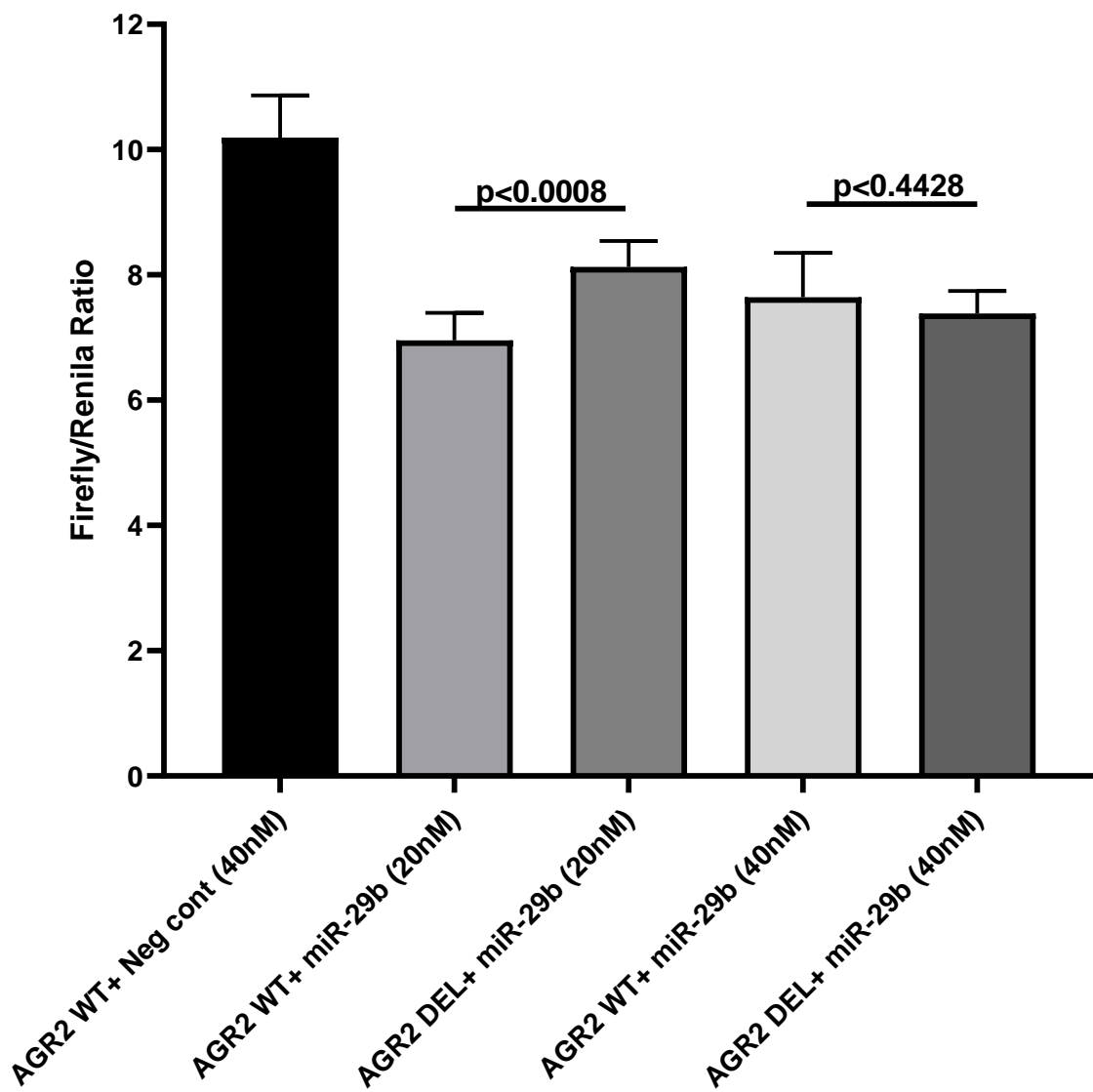

Supplement: Supplementary file 1 [file viruses-12-00713-s001.zip › Supplemental Figure S2.pdf]
